# Association of *STAT3* and *STAT4* polymorphisms with susceptibility to chronic hepatitis B virus infection and risk of hepatocellular carcinoma: a meta-analysis

**DOI:** 10.1042/BSR20190783

**Published:** 2019-06-20

**Authors:** Han Shi, Hongyan He, Suvash Chandra Ojha, Changfeng Sun, Juan Fu, Mao Yan, Cunliang Deng, Yunjian Sheng

**Affiliations:** 1 Department of Infectious Diseases, The Affiliated Hospital of Southwest Medical University, Luzhou 646000, China; 2 Experimental Teaching Center, School of Public Health of Southwest Medical University, Luzhou 646000, China

**Keywords:** HBV, HCC, Meta-analysis, single nucleotide polymorphisms, STAT3, STAT4

## Abstract

**Background:** It has been reported that polymorphisms of *signal transducer and activator of transcription (STAT*) *3* and *STAT4* might be associated with susceptibility to hepatitis B virus (HBV) infection and risk of chronic hepatocellular carcinoma (HCC). Owing to limitation of sample size and inconclusive results, we conducted a meta-analysis to clarify the association. **Methods:** We identified relevant studies by a systematic search of Medline/PubMed, Embase, Web of Science and the Cochrane Library up to 20 February 2019. The strength of the association measured by odds ratios (OR) with 95% confidence intervals (CIs) was studied. All the statistical analyses were conducted based on Review Manager 5.3 software. **Results:** A total of 5242 cases and 2717 controls from five studies were included for the *STAT3* polymorphism, 5902 cases and 7867 controls from nine studies for the *STAT4* polymorphism. Our results suggested that *STAT3* rs1053004 polymorphism was a significant risk factor of chronic HBV infection (C vs. T: OR = 1.17, 95% CI: 1.07–1.29, *P*_A_=0.0007; CC + CT vs. TT: OR = 1.38, 95% CI: 1.09–1.76, *P*_A_=0.008). Validation with all the genetic models revealed that rs7574865 polymorphism of *STAT4* gene was closely associated with chronic HBV infection (*P*_A_<0.01) and chronic hepatitis B (CHB)-related HCC (*P*_A_<0.05). Meanwhile, the authenticity of the above meta-analysis results was confirmed by trial sequential analysis (TSA). **Conclusions:** The meta-analysis showed that *STAT3* rs1053004 polymorphism may be the risk for developing chronic HBV infection but not associated with HCC. The present study also indicates that *STAT4* rs7574865 polymorphism increased the risk of chronic HBV infection and HCC.

## Introduction

Hepatitis B virus (HBV) is one of the most important human viral pathogens which causes a wide range of acute and chronic liver diseases [1]. Globally, more than 257 million people live with chronic hepatitis B (CHB) [2]. As updated on July 2018, hepatitis B surface antigen (HBsAg) seroprevalence is approximately 3.6% all over the world with highest endemicity in the African region and Western Pacific region, especially in China [3]. Following HBV persistent infection, nearly 20% patients would progress to cirrhosis and 5–10% patients would develop hepatocellular carcinoma (HCC) [4]. Chronic HBV infection remains to be a major public health problem worldwide which is known to be a major risk factor for the development of HCC. HCC is one of the most important common cancers in the world especially in Africa and East Asia. Recent studies reported that host genetic factors such as *signal transducer and activator of transcription* (*STAT*) gene polymorphisms may contribute to the risk of HBV infection and hepatic carcinogenesis.

STAT proteins are inflammatory mediators which transduce signal across the cytoplasm and function as transcription factors in the nucleus [5]. STAT pathways are crucial for regulating cell growth, differentiation, survival and death mediate cellular responses to a wide range of cytokines [6]. STAT proteins family comprises seven members, including STAT1, STAT2, STAT3, STAT4, STAT5a, STAT5b and STAT6 [7,8]. HBV infection and the carcinogenesis of HCC are complex processes that involve various modifications to a number of molecular pathways. Among STAT family protein, the STAT3 and the STAT4 play a key role in liver inflammation and cancer which have gained considerable attention. In HBV infection, dysregulated STAT3 signaling has been revealed to be involved in ineffective immune response against HBV [9,10] and the pathogenesis of liver diseases [9–11] through mediating the cytokine-mediated HBV enhancer function [9] and influencing the cytoprotective effect of hepatocyte growth factor and epidermal growth factor on CD95-mediated apoptosis and the action of cytotoxic T cells [11]. STAT4 is an important transcription factor that encodes many transcription factors transmitting signals stimulated by cytokines. It also regulates the expression of various genes as a transcription factor after it is phosphorylated, then dimerizes and translocates to the nucleus.

It has been well known that cases of liver cancer are usually diagnosed in patients with intermediate or advanced stages. Therefore, it is necessary to find solutions for diagnosing this disease at an early stage of development that could help to prevent its dissemination to advanced stages and provide timely treatment. Genome-wide association study (GWAS) is the most extensive and powerful tool among the entire genetic studies, and it has the capacity to genotype nearly several hundreds of thousands of single nucleotide polymorphisms (SNPs) throughout the entire human genome [12,13]. Other researchers employed this novel technique to investigate human complex diseases, such as cancers and congenital diseases. Recently, a number of studies have been conducted to investigate the association between the two SNPs (*STAT3*: rs1053004, rs2293152; *STAT4*: rs7574865) and the risk of HBV infection and CHB-related HCC in diverse population, but the results were contradictory and inconclusive. Up to now, there is no *STAT3* gene polymorphism meta-analysis investigating above-mentioned association. These meta-analysises about *STAT4* polymorphism [14,4,15] only clarifies the relationship with risk of HCC, without chronic HBV infection, and the samples are small. Therefore, we performed a meta-analysis to evaluate the association between the two SNPs (*STAT3, STAT4*) and HBV infection and CHB-related HCC. In addition, to minimize random errors and strengthen the robustness of our conclusions, we performed trial sequential analysis (TSA).

## Methods

### Identification of eligible studies

We carried out a systematic search in PubMed, Embase, Web of Science and the Cochrane Library, with the last search through 20 February 2019. The search was designed using the keywords: ‘*STAT3*’, ‘*STAT4*’, ‘*signal transducer and activator of transcription 3*‘, ‘*signal transducer and activator of transcription 4*’, ‘polymorphism OR SNP’, ‘HBV OR hepatitis B virus’, ‘HCC OR hepatocellular cancer OR liver carcinoma’. The reference lists of retrieved studies and recent reviews were also manually searched for further relevant studies.

### Inclusion and exclusion criteria

Studies in this meta-analysis must meet the following inclusion criteria: (i) evaluated the two SNPs (*STAT3*: rs1053004, rs2293152; *STAT4*: rs7574865) association with HBV infection or CHB-related HCC; (ii) case–control study; (iii) detailed genotype data could be acquired to calculate the odds ratios (ORs) and 95% confidence intervals (CIs) (iv) studies focusing on human beings. Exclusion criteria: (i) duplication of previous publications; (ii) case reports, basic research, review and other non-case–control studies; (iii) studies without detailed genotype data; (iv) non-English publications.

Study selection was achieved by two investigators independently (Han Shi and Hongyan He), according to the inclusion and exclusion criteria by screening the title, abstract and full-text. Any dispute was solved by discussion.

### Data extraction

The data of the eligible studies were extracted in duplicate by two investigators independently. The following data were recorded: (i) name of first author; (ii) year of publication; (iii) the characteristics of cases and controls; (iv) ethnicity; (v) genotyping methods; (vi) whether the genotypes of all component studies were tested for Hardy–Weinberg equilibrium (HWE); (vii) number of cases and controls for the two SNPs *(STAT3:* rs1053004, rs2293152; *STAT4:* rs7574865) genotypes. Two authors checked the extracted data and reached a consensus on all the data. If a dissent existed, they would recheck the original data of the included studies and have a discussion to reach consensus. If the dissent still existed, the third investigator (Yujian Sheng) was involved to adjudicate the disagreements.

### Quality assessment

Two reviewers (Han Shi and Hongyan He) independently evaluated the quality of selected studies by quality assessment scale (Supplementary Table S1) which was extracted and modified from previous studies [11,16,17]. Quality scores ranged from 0 to 15 and the studies with higher scores were considered to be of better quality. High quality study was defined by scores more than 9. Disagreements were resolved by discussion.

### TSA

The reliability and authenticity of the results of meta-analysis will be verified by TSA, provide references for future clinical studies. TSA parameter setting: type I error probability of 5%, type II error probability of 35%, and risk ratio reduction (RRR) of 15% to calculated the Require Information Size (RIS) [18]. TSA was not performed for the association in *STAT3* (rs2293152) study owing to the limited number of included studies.

### False-positive report probability analysis

The significant findings of meta-analysis will be verified by false-positive report probability (FPRP) [19,20]. We set the FPRP threshold to 0.2 assigned a prior probability of 0.1 and detect an OR of 0.67/1.50 (protective/risk effects) for an association with genotypes under investigation. The significant result with an FPRP value less than 0.2 was considered a noteworthy finding. All the calculations to derive FPRP were performed with the Excel spreadsheet released by Wacholder et al. [19].

### Statistical analysis

Publication bias was solved by symmetrical funnel Begg’s plot analysis. Crude ORs and corresponding 95% CIs were calculated to investigate the association strength between *STAT* polymorphisms and chronic HBV infection and CHB-related HCC. Pooled ORs were obtained from combination of single studies by allelic comparison (rs1053004: C vs. T; rs2293152: G vs. C; rs7574865: G vs. T), dominant model (rs1053004: CC + CT vs. TT; rs2293152: GG + GC vs. CC; rs7574865: GG + GT vs. TT), recessive model (rs1053004: CC vs. CT + TT; rs2293152: GG vs. GC + CC; rs7574865: GG vs. GT + TT). We used chi-square-based Q-test [21] and the *I* square (*I^2^*) index [22] to check the heterogeneity among different studies. In the absence of significant statistic heterogeneity (*P*-value more than 0.10), we used the fixed-effects model, otherwise, the random-effects model. The overall effect was tested using z scores calculated by Fisher’s z transformation with significance set at *P*<0.05. Sensitivity analysis was also performed to evaluate the effect of each study on the combined ORs by omitting each study in each turn and excluding the HWE-violating studies to check the robustness of the result. Besides, subgroup analyses were stratified by ethnicity, and publication bias was checked. If study does not describe the HWE, we calculated it by Chi-square test. *P*-value, less than 0.05 was considered to be a state of disequilibrium. All statistical analyses were performed with Review Manager 5.3 software.

## Results

### Characteristics of included studies

**Figure 1:**
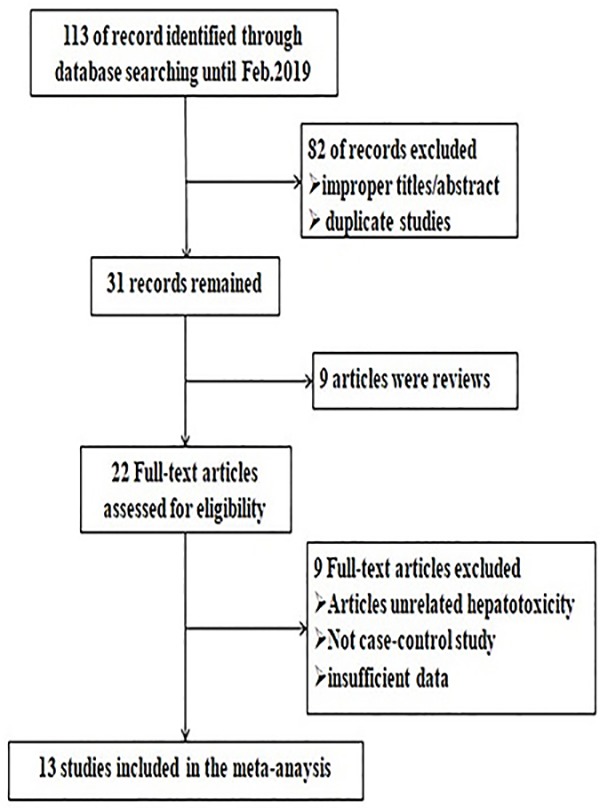
Chart of the literature search and selection process

A total of 113 studies were acquired from Medline/PubMed, Embase, Web of Science and the Cochrane Library databases. The literature selection process was shown in Figure 1. Finally, 13 studies were selected describing the strength of the postulated genetic associations of *STAT3* and *STAT4* polymorphisms with chronic HBV infection and CHB-related HCC. There were four studies involving 2928 patients and 1518 controls for *STAT3* rs1053004, two studies including 2314 patients and 1199 controls for *STAT3* rs2293152, nine studies including 5902 cases and 7867 controls for *STAT4* rs7574865. Among these studies, Xie et al. [23] described both *STAT3* rs1053004 and *STAT3* rs2293152. Chanthra et al. [24] described both *STAT3* rs2293152 and *STAT4* rs7574865. For *STAT3* rs1053004, three studies [23,25,26] were from Southeast Asian (China, Thailand) and the other one from west Asian (Iran) populations [27]. For *STAT3* rs2293152, two studies were carried out in China [23] and Thailand separately [24]. At last, nine studies were identified of *STAT4* rs7574865, of which eight studies came from Asian [4,24,28–33] and one study came from Caucasian [34] population.

Quality assessment of all the included studies showed that only study conducted by Fatemipour et al. [27] scored less than 9 points which was regarded as study of low quality. The rest of studies were of high quality ranging from 10 to 14. Most studies indicated that the frequencies distribution of genotypes in the controls were consistent with HWE. Deviations from HWE were observed only in Fatemipour et al. [27]. Characteristics of enrolled studies were assigned to the structured form (Table 1).

**Table 1:** Characteristics of studies included in the meta-analysis

SNP	Author	Year	Country	Ethnicity	HWE	CHB			HCC			HC			NC			Scores
						Gene type (*n*)												
*STAT3* (rs1053004)						TT	TC	CC	TT	TC	CC	TT	TC	CC	TT	TC	CC	
	Xie et al. [23]	2013	China	Han	Y	385	451	130	411	458	140	453	400	142	/	/	/	14
	Chanthra et al. [25]	2015	Thailand	Thai	Y	73	127	33	55	107	49	77	99	30	/	/	/	13
	Fatemipour et al. [27]	2016	Iran	Iran	N	20	8	22	10	18	5	32	4	14	/	/	/	6
	Li et al. [26]	2018	China	Han	Y	108	103	28	82	82	23	88	76	5	42	42	14	13
*STAT3* (rs2293152)						GG	GC	CC	GG	GC	CC	GG	GC	CC	GG	GC	CC	
	Xie et al. [23]	2013	China	Han	Y	198	466	245	252	496	265	215	500	294	/	/	/	14
	Chanthra et al. [24]	2015	Thailand	Thai	Y	60	95	45	56	97	39	53	95	42	/	/	/	13
*STAT4* (rs7574865)						GG	GT	TT	GG	GT	TT	GG	GT	TT	GG	GT	TT	
	Chen et al. [28]	2013	China	Han	Y	370	327	75	249	217	35	/	/	/	/	/	/	13
	Clark et al. [29]	2013	Vietnam	Vietnamese	Y	86	92	28	117	107	20	/	/	/	/	/	/	12
	Kim et al. [30]	2014	Korea	Korean	Y	334	261	63	160	103	20	1293	1251	306	/	/	/	11
	Liao et al. [4]	2014	China	Han	Y	190	204	46	104	93	25	97	113	27	181	157	53	14
	Liao et al. [31]	2015	China	Tibetans	Y	194	189		/	/	/		/	/	/	209	268	14
		2015	China	Uygurs	Y	103	95		/	/	/		/	/	/	91	119	14
	Chanthra et al. [24]	2015	Thailand	Thai	Y	83	93	24	87	86	19	62	100	28	/	/	/	13
	Chen et al. [32]	2015	China	Han	Y	/	/	/	257	211	40	1298	1333	343	/	/	/	11
	Lu et al. [33]	2015	China	Han	Y	77	95	15	45	30	3	/	/	/	114	132	37	10
	El Sharkawy et al. [34]	2018	Sydney	Caucasian	Y	546	252	32	/	/	/	147	93	15	/	/	/	13

Abbreviations: HC, healthy control; NC, natural clearance subject.

**Table 2:** Meta-analysis of the association between *STAT3* polymorphisms and chronic HBV infection and CHB-related HCC risk

Case/control	SNP	Included studies		Genetic model	OR	95% CI	*I^2^* (%)	*P*_H_	Z	*P*_A_
CHB vs. HC+NC	rs1053004 (T>C)	Overall	4	Allelic effect (C vs. T)	1.17	1.07–1.29	48	0.13	3.39	0.0007
			4	Dominant effect (CC+TC vs. TT)	1.38	1.09–1.76	56	0.08	2.63	0.008
			4	Recessive effect (CC vs. TC+TT)	1.1	0.91–1.31	44	0.15	0.99	0.32
		Southeast Asian	3	Allelic effect (C vs. T)	1.15	1.05–1.27	0	0.43	2.98	0.003
			3	Dominant effect (CC+TC vs. TT)	1.26	1.11–1.43	0	0.61	3.49	0.0005
			3	Recessive effect (CC vs. TC+TT)	1.23	0.85–1.77	62	0.07	1.08	0.28
	rs2293152 (C>G)	Overall	2	Allelic effect (G vs. C)	1.09	0.99–1.20	0	0.73	1.72	0.08
			2	Dominant effect (GG+GC vs. CC)	1.12	0.96–1.32	0	0.7	1.46	0.14
			2	Recessive effect (GG vs. GC+CC)	1.12	0.95–1.32	0	0.86	1,35	0.18
CHB related HCC vs. CHB without HCC	rs1053004 (T>C)	Overall	4	Allelic effect (C vs. T)	1.04	0.93–1.15	48	0.12	0.64	0.52
			4	Dominant effect (CC+TC vs. TT)	1.03	0.89–1.19	0	0.49	0.37	0.71
			4	Recessive effect (CC vs. TC+TT)	0.98	0.58–1.67	76	0.006	0.06	0.95
		Southeast Asian	3	Allelic effect (C vs. T)	1.05	0.94–1.17	50	0.14	0.86	0.39
			3	Dominant effect (CC+TC vs. TT)	1.02	0.87–1.18	0	0.43	0.22	0.82
			3	Recessive effect (CC vs. TC+TT)	1.16	0.94–1.43	53	0.12	1.37	0.17
	rs2293152 (C>G)	Overall	2	Allelic effect (G vs. C)	1.07	0.95–1.20	0	0.75	1.18	0.24
			2	Dominant effect (GG+GC vs. CC)	1.06	0.88–1.27	0	0.74	0.57	0.57
			2	Recessive effect (GG vs. GC+CC)	1.14	0.94–1.38	0	0.39	1.36	0.17

Abbreviations: *P*_H_, *P*-value of heterogeneity; *P*_A_, adjusted

*P-*value (*P*_A_<0.05 means statistically significant).

**Table 3:** Meta-analysis of the association between *STAT4* polymorphisms and chronic HBV infection and CHB-related HCC risk

Case/Control	SNP	Included studies		Genetic model	OR	95% CI	*I^2^* (%)	*P*_H_	Z	*P*_A_
CHB vs. HC	rs7574865 (G>T)	Overall	6	Allelic effect (G vs T)	1.23	1.15–1.32	8	0.37	5.97	<0.00001
			6	Dominant effect (GG+GT vs TT)	1.38	1.18–1.61	0	0.61	4.04	<0.0001
			8	Recessive effect (GG vs GT+TT)	1.29	1.19–1.41	0	0.43	6,04	<0.00001
		Asian	5	Allelic effect (G vs T)	1.22	1.14–1.32	17	0.31	5.5	<0.00001
			5	Dominant effect (GG+GT vs TT)	1.36	1.16–1.60	0	0.51	3.81	0.0001
			7	Recessive effect (GG vs GT+TT)	1.28	1.18–1.40	9	0.36	5.6	<0.00001
CHB related HCC vs. CHB without HCC	rs7574865 (G>T)	Overall	6	Allelic effect (G vs T)	1.18	1.07–1.31	0	0.51	3.26	0.001
			6	Dominant effect (GG+GT vs TT)	1.26	1.04–1.53	0	0.56	2.31	0.02
			6	Recessive effect (GG vs GT+TT)	1.2	1.06–1.37	0	0.49	2.78	0.005

Abbreviations: *P*_A_, adjusted; *P*_H_, *P*-value of heterogeneity.

*P-*value (*P*_A_<0.05 means statistically significant).

### *STAT3* (rs1053004, rs2293152) polymorphism meta-analysis (Table 2)

#### *STAT3* polymorphism and susceptibility to chronic HBV infection

**Figure 2:**
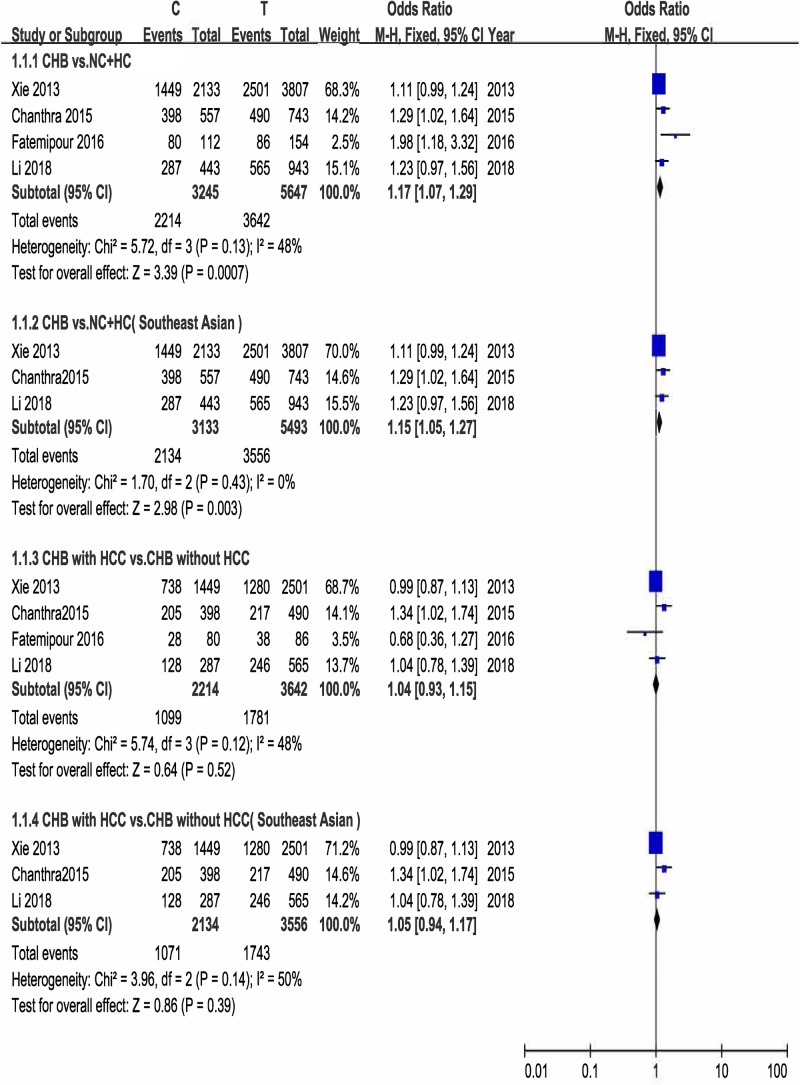
Forest plot of allele comparison of *STAT3* rs1053004 for CHB susceptibility and CHB-related HCC risk

**Figure 3:**
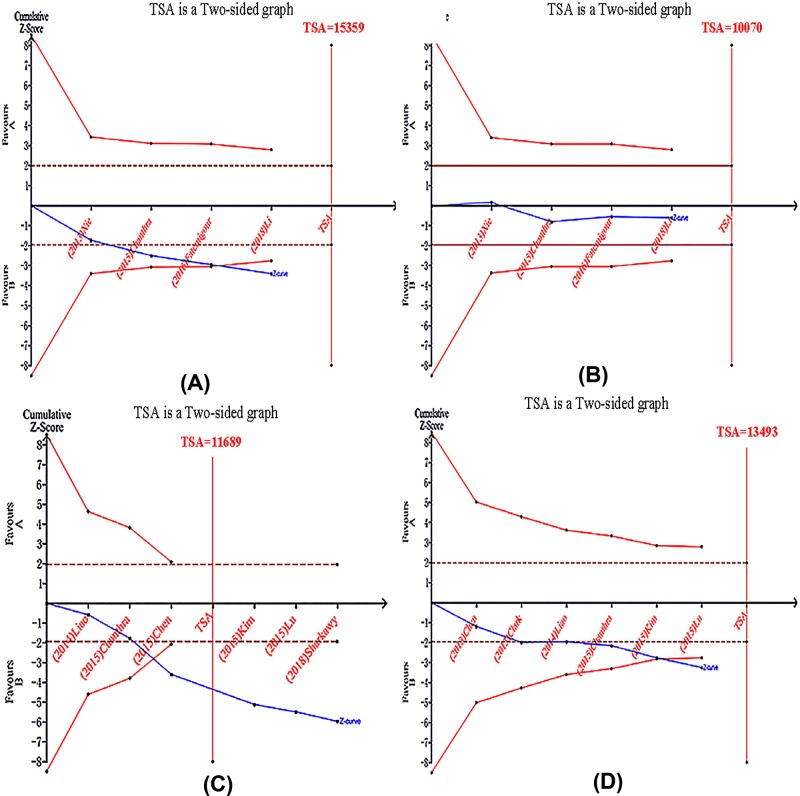
TSA for *STAT* polymorphism under the allele contrast model (**A**) Chronic HBV infection susceptibility in *STAT3* rs1053004. (**B**) Risk of CHB-related HCC in *STAT3* rs1053004. (**C**) Chronic HBV infection susceptibility in *STAT4* rs7574865. (**D**) Risk of CHB-related HCC in *STAT4* rs7574865.

We first analyzed the association between *STAT3* rs1053004 polymorphism and susceptibility to HBV infection. Significant heterogeneity was identified only in recessive model by Q-test and I-squared statistic, and random-effects model was used. Fixed-effects model was used in other two genetic models. According to the data, *STAT3* rs1053004 genotype might increase the risk of chronic HBV infection (C vs. T: OR = 1.17, 95% CI: 1.07–1.29, *P*_A_=0.0007; CC + CT vs. TT: OR = 1.38, 95% CI: 1.09–1.76, *P*_A_=0.008; CC vs. CT + TT: OR = 1.10, 95% CI: 0.91–1.31, *P*_A_=0.32). The C allele may be a risk factor. The forest plots are shown in Figure 2 and Supplementary Figures S1 and S2. We performed a TSA, Z-curve crossed TSA boundary even if the sample size did not reach RIS, which confirmed the certain result (Figure 3A). Subgroup analysis was conducted according to ethnicity, after deleting the West Asian study (Fatemipour et al. (2016) [27]), the results were the same (C vs. T: OR = 1.15, 95% CI: 1.05–1.27, *P*_A_=0.003; CC + CT vs. TT: OR = 1.26, 95% CI: 1.11–1.43, *P*=0.0005, CC vs. CT + TT: OR = 1.23, 95% CI: 0.85–1.77, *P*_A_=0.28). The forest plots are shown in Figure 2 and Supplementary Figures S3 and S4. In *STAT3* rs2293152 study, there was no significant heterogeneity in any of the genetic models. Therefore, fixed-effects model was used. But *STAT3* rs2293152 seemed not to be correlated with HBV infection (G vs. C: OR = 1.09, 95% CI: 0.99–1.20, *P*_A_=0.08; GG + GC vs. CC: OR = 1.12, 95% CI: 0.96–1.32, *P*_A_=0.14; GG vs. GC + CC: OR = 1.12, 95% CI: 0.95–1.32, *P*_A_=0.18). The forest plots were shown in Supplementary Figures S5–S7.

#### *STAT3* polymorphism and CHB-related HCC

In *STAT3* rs1053004 study, random-effects model was used in the recessive model due to presence of heterogeneity, and fixed-effects model was used in the other two models. We observed no significant connection between *STAT3* rs1053004 polymorphism and CHB-related HCC risk (C vs. T: OR = 1.04, 95% CI: 0.93–1.15, *P*_A_=0.52; CC + CT vs. TT: OR = 1.03, 95% CI: 0.89–1.19, *P*_A_=0.71; CC vs. CT + TT: OR = 0.98, 95% CI: 0.58–1.67, *P*_A_=0.95). The forest plots are shown in Figure 2 and Supplementary Figures S3 and S4. TSA was taken, the result of TSA indicated that the sample size of effectiveness did not achieve either TSA Boundary or RIS. It means the result showed no difference was statistically significant, studies of high quality and large samples are needed (Figure 3B). After deleting the study (Fatemipour et al. (2016) [27]), the results were not changed in subgroup analysis (C vs. T: OR = 1.05, 95% CI: 0.94–1.17, *P*_A_=0.39; CC + CT vs. TT: OR = 1.02, 95% CI: 0.8–1.18, *P*_A_=0.82; CC vs. CT + TT: OR = 1.16, 95% CI: 0.94–1.43, *P*_A_=0.17). The forest plots are shown in Figure 2 and Supplementary Figures S2 and S3. Likewise, we found no significant connection between *STAT3* rs2293152 polymorphism and CHB-related HCC risk either (G vs. C, OR = 1.07, 95% CI: 0.95–1.20, *P*_A_=0.24; GG + GC vs. CC: OR = 1.06, 95% CI: 0.88–1.27, *P*_A_= 0.57; GG vs. GC + CC: OR = 1.14, 95% CI: 0.94–1.38, *P*_A_=0.17). The forest plots were shown in Supplementary Figures S5–S7. However, a trend of increase risk could be seen.

### *STAT4* (rs7574865) polymorphism meta-analysis (Table 3)

#### *STAT4* polymorphism and susceptibility to chronic HBV infection

**Figure 4:**
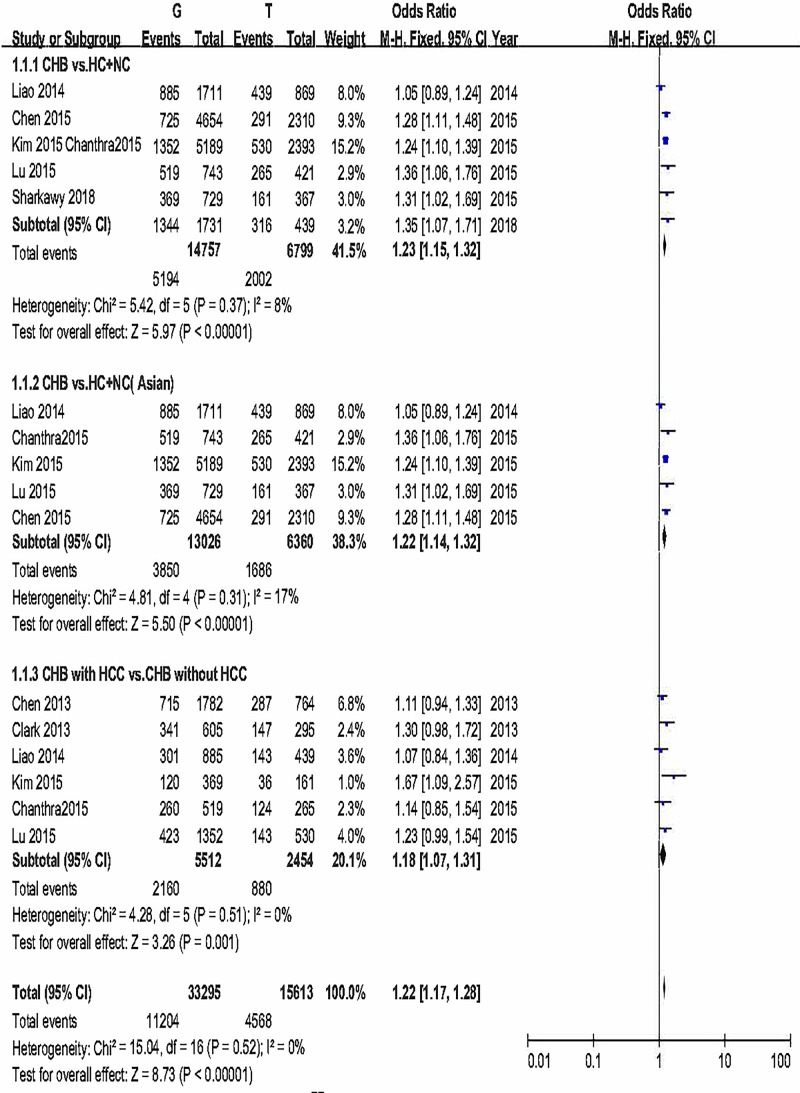
Forest plot of allele comparison of *STAT4* rs7574865 For CHB susceptibility and CHB-related HCC risk.

Additionally, we carried out a comprehensive meta-analysis of *STAT4* rs7574865 polymorphism. Because of no significant heterogeneity in any of the genetic models, fixed-effects model was used. Significant statistical differences were identified in all the genetic models (G vs. T: OR = 1.23, 95% CI: 1.15–1.32, *P*_A_<0.00001; GG + GT vs. TT: OR = 1.38, 95% CI: 1.18–1.61, *P*_A_<0.0001; GG vs. GT + TT: OR = 1.29, 95% CI: 1.19–1.41, *P*_A_<0.00001). The forest plots are shown in Figure 4 and Supplementary Figures S8 and S9. Above results indicated that *STAT4* rs7574865 genotype might significantly increase the risk of chronic HBV infection. Individuals carrying at least one G allele (GG or GT genotypes) for rs7574865 seemed to have higher risk of acquiring chronic HBV infection in comparison with TT genotype. Next, subgroup analysis was conducted according to ethnicity. Seven out of the eight studies were carried out in Asian population. Significant statistical differences were identified in all the genetic models (G vs. T: OR = 1.22, 95% CI: 1.14–1.32, *P*_A_<0.00001; GG + GT vs. TT: OR = 1.36, 95% CI: 1.16–1.60, *P*_A_=0.0001; GG vs. GT + TT: OR = 1.28, 95% CI: 1.18–1.40, *P*_A_<0.00001). The forest plots are shown in Figure 4 and Supplementary Figures S8 and S9. TSA was executed, the TSA result suggested that z-curve crossed the trial sequential monitoring boundary before reaching the required information size. So the cumulative evidence results were reliability and no further evidence is needed to verify the conclusions (Figure 3C).

#### *STAT4* polymorphism and CHB-related HCC

At last, we appraised the correlation between *STAT4* rs7574865 and CHB-related HCC. We did not find significant heterogeneity, so fixed-effects were used. The overall results suggested that *STAT4* rs7574865 still correlated with CHB-related HCC (G vs. T: OR = 1.18, 95% CI: 1.07–1.31, *P*_A_=0.001; GG + GT vs. TT: OR = 1.26, 95% CI: 1.04–1.53, *P*_A_=0.02; GG vs. GT + TT: OR = 1.20, 95% CI: 1.06–1.37, *P*_A_=0.005). The forest plots are shown in Figure 4, Supplementary Figures S8 and S9. TSA was performed. The result of TSA indicated that although the sample size of effectiveness did not achieve RIS, a certain conclusion is obtained in advance owing to the sample size reach the TSA Boundary (Figure 3D). No more tests required.

### FPRP analysis

Results of association between genetic variants and diseases may be subjected to false positivity. Tables 4 and 5 showed the FPRP values for our positive results using different prior probability levels. FPRP value below 0.2 was noteworthy. On research of associations between *STAT3* polymorphism and HBV infection susceptibility, when prior probability of 0.1 was adopted, significant association for rs1053004 T > C (C vs. T, CC + TC vs. TT) was verified to be noteworthy (FPRP < 0.2). None of positive results of sensitivity analysis for rs2293152 C > G was considered noteworthy (FPRP > 0.2). All results for rs7574865 G > T were deserving of attention (FPRP < 0.2) (Table 4). On research of associations between *STAT3* polymorphism and CHB-related HCC risk, the FPRP values were all >0.20, showed that these significant associations were not noteworthy. We finally calculated the FPRP values about *STAT4* polymorphism with CHB-related HCC risk, the FPRP values were all <0.20, suggesting that these significant associations were noteworthy (Table 5).

**Table 4:** FPRP values for associations between *STAT3, STAT4* polymorphism and chronic HBV infection

SNP		Genetic model	OR	95% CI	*P*	Power	Prior probability				
							0.25	0.1	0.01	0.001	0.0001
*STAT3* rs1053004 (T>C)	Overall	Allelic effect (C vs. T)	1.17	1.07–1.29	0.001623	1.000	0.005	0.014	0.138	0.619	0.942
		Dominant effect (CC+TC vs. TT)	1.38	1.09–1.76	0.009448	0.749	0.036	0.102	0.555	0.926	0.992
		Recessive effect (CC vs. TC+TT)	1.10	0.91–1.31	0.284978	1.000	0.461	0.72	0.966	0.997	1.000
	Southeast Asian	Allelic effect (C vs. T)	1.15	1.05–1.27	0.005782	1.000	0.017	0.049	0.364	0.852	0.983
		Dominant effect (CC+TC vs. TT)	1.26	1.11–1.43	0.000345	0.997	0.001	0.003	0.033	0.257	0.776
		Recessive effect (CC vs. TC+TT)	1.23	0.85–1.77	0.264937	0.857	0.481	0.736	0.968	0.997	1.000
*STAT3* rs2293152 (C>G)	Overall	Allelic effect (G vs. C)	1.09	0.99–1.20	0.078947	1.000	0.191	0.415	0.887	0.987	0.999
		Dominant effect (GG+GC vs. CC)	1.12	0.96–1.32	0.176402	1.000	0.346	0.614	0.946	0.994	0.999
		Recessive effect (GG vs. GC+CC)	1.12	0.95–1.32	0.176402	1.000	0.346	0.614	0.946	0.994	0.999
*STAT4* rs7574865 (G>T)	Overall	Allelic effect (G vs. T)	1.23	1.15–1.32	0.000000	1.000	0.000	0.000	0.000	0.000	0.000
		Dominant effect (GG+GT vs. TT)	1.38	1.18–1.61	0.000042	0.855	0.000	0.000	0.005	0.047	0.330
		Recessive effect (GG vs. GT+TT)	1.29	1.19–1.41	0.000000	1.000	0.000	0.000	0.000	0.000	0.000
	Asian	Allelic effect (G vs. T)	1.22	1.14–1.32	0.000001	1.000	0.000	0.000	0.000	0.001	0.007
		Dominant effect (GG+GT vs. TT)	1.36	1.16–1.60	0.000209	0.881	0.001	0.002	0.023	0.191	0.703
		Recessive effect (GG vs. GT+TT)	1.28	1.18–1.40	0.000000	1.000	0.000	0.000	0.000	0.000	0.001

Abbreviations; CI, confidence interval; OR, odds ratio.

*P*, Chi-square test was adopted to calculate the genotype frequency distributions.

Power, Statistical power was calculated using the number of observations in the subgroup and the OR and *P* values in this table.

**Table 5:** FPRP values for associations between *STAT3, STAT4* polymorphism and CHB-related HCC risk

SNP		Genetic model	OR	95% CI	*P*	Power	Prior probability				
							0.25	0.1	0.01	0.001	0.0001
*STAT3* rs1053004 (T>C)	Overall	Allelic effect (C vs. T)	1.04	0.93–1.15	0.444517	1.000	0.571	0.800	0.978	0.998	1.000
		Dominant effect (CC+TC vs. TT)	1.03	0.89–1.19	0.688252	1.000	0.674	0.861	0,986	0.999	1.000
		Recessive effect (CC vs. TC+TT)	0.98	0.58–1.67	0.940781	0.922	0.754	0.902	0.990	0.999	1.000
	Southeast Asian	Allelic effect (C vs. T)	1.05	0.94–1.17	0.376856	1.000	0.531	0.772	0.974	0.997	1.000
		Dominant effect (CC+TC vs. TT)	1.02	0.87–1.18	0.789955	1.000	0.703	0.877	0.987	0.999	1.000
		Recessive effect (CC vs. TC+TT)	1.16	0.94–1.43	0.164472	0.992	0.332	0.599	0.943	0.994	0.999
*STAT3* rs2293152 (C>G)	Overall	Allelic effect (G vs. C)	1.07	0.95–1.20	0.247465	1.000	0.426	0.690	0.961	0.996	1.000
		Dominant effect (GG+GC vs. CC)	1.06	0.88–1.27	0.527480	1.000	0.613	0.826	0.981	0.998	1.000
		Recessive effect (GG vs. GC+CC)	1.14	0.94–1.38	0.178886	0.998	0.350	0.617	0.947	0.994	0.999
*STAT4* rs7574865 (G>T)	Overall	Allelic effect (G vs. T)	1.18	1.07–1.31	0.001909	1.000	0.006	0.017	0.159	0.656	0.950
		Dominant effect (GG+GT vs. TT))	1.26	1.04–1.53	0.019645	0.961	0.058	0.155	0.669	0.953	0.995
		Recessive effect (GG vs. GT+TT)	1.20	1.06–1.37	0.006992	1.000	0.021	0.059	0.409	0.875	0.986

Abbreviations; CI, confidence interval; OR, odds ratio.

*P*, Chi-square test was adopted to calculate the genotype frequency distributions.

Power, Statistical power was calculated using the number of observations in the subgroup and the OR and *P* values in this table.

### Sensitivity analysis

In order to check the influence by the individual study on the overall ORs, we deleted each study once in every genetic model. The sensitivity analysis demonstrated that the *STAT3* rs1053004 and *STAT4* rs7574865 ORs were not statistically influenced, which validated the stability of our data. As for *STAT3* rs1053004, sensitivity analysis was also carried out by excluding Fatemipour et al.’s study [27], which was HWE-violating and low quality. However, the results still had not been changed. Subgroup analysis was conducted according to ethnicity, and the heterogeneity decreased obviously. Inconsistency between the two ethnicities can be explained by the possibility that different ethnic groups live with multiple life styles and environmental factors and thus yield diverse gene–environment interactions [35].

### Publication bias

**Figure 5:**
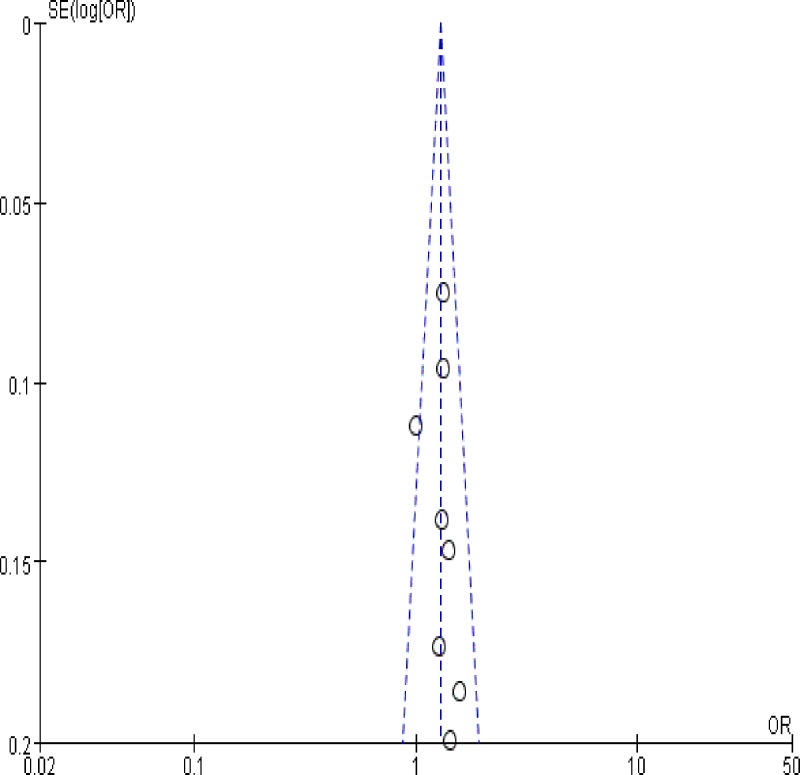
Begg’s funnel plot to detect publication bias analysis for *STAT4* rs7574865 polymorphism under the recessive contrast model

Based on symmetrical funnel plots analysis, no remarkable asymmetry in the distribution of scattered points is observed (Figure 5). No evidence of publication bias was found. No funnel plot was performed for the association in *STAT3* (rs1053004, rs2293152) study owing to the limited number of included studies.

## Discussion

Recently, several studies explored the relationship between *STAT3, STAT4* polymorphisms and the risk of HCC. However, the results of the studies have been inconclusive or inconsistent. In order to clarify this obscure correlation, we made this meta-analysis. Since the minor allele frequencies (MAFs) provide resemblance not only between healthy control (HC) and natural clearance (NC) but also between CHB without HCC and CHB-related HCC; hence, we divided all the patients into two groups: control (NC + HC) and case (CHB without HCC + CHB-related HCC) to explore the relationship between *STAT3, STAT4* polymorphisms and chronic HBV infection susceptibility. When we researched the relationship between *STAT3, STAT4* polymorphisms and HCC risk, CHB without HCC as control group and CHB-related HCC as case group.

*STAT3* is a transcription factor which in humans is encoded by the *STAT3* gene. *STAT3* has been shown to play pivotal role in the transcription of genes important for inflammation, survival, proliferation and invasion of HCC [8,36]. *STAT3* has been shown to be involved in the enhanced Th17 response in acute-on-chronic liver failure (ACLF) associated with HBV infection [37] and the HBV reactivation in liver after radiotherapy [38]. Moreover, previous studies indicated that *STAT3* can be activated by the X protein of HBV and activated *STAT3* can also bind with the HBV enhancer 1 to activate gene expression, suggesting the interplay between *STAT3* and X protein in promoting HCC development [9]. To our surprise, we only found that *STAT3* rs1053004 on C allele is the risk factor for developing chronic HBV infection. However, we did not find significant association between *STAT3* rs2293152 and chronic HBV infection. We also observed both *STAT3* rs1053004 and *STAT3* rs2293152 did not correlate with CHB-related HCC. Neither allele frequency nor genotype distribution showed significant association with the risk of CHB-related HCC which was not consistent with Xie et al. [23] and Fatemipour et al. [27]. Following reasons could explain this inconsistent. First, many factors would be components in the initiation, promotion and progression of the CHB to HCC and genetic variation is only one of the factor. Next, the inconsistency could be due to the sample size and sampling error in the study which were confirmed by TSA and FPRP. Well-designed studies with larger sample size and more ethnic groups are required to validate the associations. In this process, significant heterogeneity was found in *STAT3* rs1053004 study in recessive model. However, when we restricted the ethnicity to Southeast Asian, there was no heterogeneity in existence, suggesting that ethnicity to some extent contributed to the source of heterogeneity. Though heterogeneity existed, our results remained stable.

In addition to STAT3 protein, STAT4 is also a member of STAT family proteins [8]. STAT4 is a latent cytosolic factor that encodes many transcription factors transmitting signals stimulated by cytokines (i.e., IFNs, IL-12 and IL-23) [39]. STAT4 serine phosphorylation is essential for IL-12-induced IFN-γ production, and STAT4-related signaling regulates cellular activities of Th1-type T cells by triggering transcription of potent genes, such as IFN-γ [40–42]. The above results have been confirmed in these studies of Chanthra et al. [24], Chen et al. [32], Lu et al. [33] and El Sharkawy et al. [34]. Especially, Chanthra et al. [24] and Zhang et al. [15] pointed out G allele was a risk allele for HCC development. However, Clark et al. [29], Kim et al. [30] and Chen et al. [32] replication result for rs7574865 showed no association with risk of HCC. One meta-analysis [4] reported that *STAT4* rs7574865 seemed not to correlate with HBV susceptibility, and it seemed rather ambiguous in its role on HCC development at present. Because we included more studies in our meta-analysis, we not only found the significantly association between *STAT4* rs7574865 and chronic HBV infection but also between *STAT4* rs7574865 and CHB-related HCC in three genetic models. Again, G allele of *STAT4* rs7574865 was the risk factor. Similar results were found in subgroup analysis by ethnicity and sensitivity analysis.

Our study assessed the authenticity of the meta-analysis results by TSA and FPRP verified. Except the outcome of relationship between *STAT3* polymorphism and CHB-related HCC, other results of TSA were reliable and had statistically significant.

Our meta-analysis has several strengths. As per our knowledge, this is the first meta-analysis about *STAT3* polymorphism association with chronic HBV infection susceptibility and CHB-related HCC. Based on the previous studies of the relationship between the SNPs and the risk of HCC, we also added the analysis about the relationship between the two SNPs and susceptibility to chronic HBV infection. Compared with the former meta-analysis about *STAT4* gene, more studies were included, and supplementary analysis including subgroup, TSA and FPRP analysis were performed. Moreover, most of included studies had acceptable quality (scored at least 9) and only one study was low quality. In spite of the considerable efforts to explore the possible relationship between the two SNPs, some limitations should be considered. First, the number of enrolled studies for each polymorphism still was fewer, particularly the studies analyzing the *STAT3* rs2293152 polymorphism (only two case–control studies). Second, limiting the study to English language articles may have potentially led to a language bias. Last, the analysis was only based on genotyping data, and we were unable to explore the effects of gene, gene interactions or gene-environment interactions. Human genes are unlikely to work alone during disease development.

## Conclusions

Our results suggested that *STAT3* rs1053004 polymorphism may be associated with susceptibility of chronic HBV infection, but is not associated with increased risk of CHB-related HCC. *STAT3* rs2293152 polymorphism may not be associated with susceptibility of HBV infection and CHB-related HCC. Meanwhile, our study provided convincing evidence of the genetic involvement of *STAT4* rs7574865 polymorphism in chronic HBV infection and CHB-related HCC. Its benefits to clinicians and researchers to improve the efficacy of multi-layers of prevention, precise approaches of diagnosis and treatment.

## Supporting information

**Supplementary Figure S1:** 

**Supplementary Figure S2:** 

**Supplementary Figure S3:** 

**Supplementary Figure S4:** 

**Supplementary Figure S5:** 

**Supplementary Figure S6:** 

**Supplementary Figure S7:** 

**Supplementary Figure S8:** 

**Supplementary Figure S9:** 

**Supplementary Table S1:** Score of quality assessment

## Abbreviations

CHB: chronic hepatitis B
CI: confidence interval
FPRP: false-positive report probability
HBV: hepatitis B virus
HC: healthy control
HCC: hepatocellular carcinoma
HWE: Hardy–Weinberg equilibrium
IFN: interferon
IL: interleukin
NC: natural clearance
OR: odds ratio
RIS: Require Information Size
SNP: single nucleotide polymorphism
STAT: *signal transducer and activator of transcription*
TSA: trial sequential analysis

## References

[1] Blumberg B.S. (2006) The curiosities of hepatitis B virus: prevention, sex ratio, and demography. Proc. Am. Thorac. Soc. 3, 14–20 10.1513/pats.200510-108JH 16493147

[2] Centres for Disease Control and Prevention (2008) World Hepatitis Day report. http://wwwcdcgov/features/worldhepatitisday/indexhtml

[3] Schweitzer A., Horn J., Mikolajczyk R.T., Krause G. and Ott J.J. (2015) Estimations of worldwide prevalence of chronic hepatitis B virus infection: a systematic review of data published between 1965 and 2013. Lancet 386, 1546–1555 10.1016/S0140-6736(15)61412-X 26231459

[4] Liao Y., Cai B., Li Y., Chen J., Tao C., Huang H. (2014) Association of HLA-DP/DQ and STAT4 polymorphisms with HBV infection outcomes and a mini meta-analysis. PLoS ONE 9, e111677 10.1371/journal.pone.0111677 25365208 PMC4218798

[5] Yu H., Pardoll D. and Jove R. (2009) STATs in cancer inflammation and immunity: a leading role for STAT3. Nat. Rev. Cancer 9, 798–809 10.1038/nrc2734 19851315 PMC4856025

[6] Rane S.G. and Reddy E.P. (2000) Janus kinases: components of multiple signaling pathways. Oncogene 19, 5662–5679 10.1038/sj.onc.1203925 11114747

[7] Yu H. and Jove R. (2004) The STATs of cancer–new molecular targets come of age. Nat. Rev. Cancer 4, 97–105 10.1038/nrc1275 14964307

[8] Subramaniam A., Shanmugam M.K., Perumal E., Li F., Nachiyappan A., Dai X. (2013) Potential role of signal transducer and activator of transcription (STAT)3 signaling pathway in inflammation, survival, proliferation and invasion of hepatocellular carcinoma. Biochim. Biophys. Acta 1835, 46–60 23103770 10.1016/j.bbcan.2012.10.002

[9] Waris G. and Siddiqui A. (2002) Interaction between STAT-3 and HNF-3 leads to the activation of liver-specific Hepatitis B virus enhancer 1 function. J. Virol. 76, 2721–2729 10.1128/JVI.76.6.2721-2729.2002 11861839 PMC135980

[10] Koeberlein B., zur Hausen A., Bektas N., Zentgraf H., Chin R., Nguyen L.T. (2010) Hepatitis B virus overexpresses suppressor of cytokine signaling-3 (SOCS3) thereby contributing to severity of inflammation in the liver. Virus Res. 148, 51–59 10.1016/j.virusres.2009.12.003 20005910

[11] Barreiros A.P., Sprinzl M., Rosset S., Hohler T., Otto G., Theobald M. (2009) EGF and HGF levels are increased during active HBV infection and enhance survival signaling through extracellular matrix interactions in primary human hepatocytes. Int. J. Cancer 124, 120–129 10.1002/ijc.23921 18844210

[12] Ku C.S., Loy E.Y., Pawitan Y. and Chia K.S. (2010) The pursuit of genome-wide association studies: where are we now? J. Hum. Genet. 55, 195–206 10.1038/jhg.2010.19 20300123

[13] Kilpinen H. and Dermitzakis E.T. (2012) Genetic and epigenetic contribution to complex traits. Hum. Mol. Genet. 21, R24–R28 10.1093/hmg/dds383 22976472

[14] Zhao X., Jiang K., Liang B. and Huang X. (2015) STAT4 gene polymorphism and risk of chronic hepatitis B-induced hepatocellular carcinoma. Cell Biochem. Biophys. 71, 353–357 10.1007/s12013-014-0205-0 25178516

[15] Zhang L., Xu K., Liu C. and Chen J. (2017) Meta-analysis reveals an association between signal transducer and activator of transcription-4 polymorphism and hepatocellular carcinoma risk. Hepatol Res. 47, 303–311 10.1111/hepr.12733 27126090

[16] Camargo M.C., Mera R., Correa P., Peek R.M. Jr, Fontham E.T., Goodman K.J. (2006) Interleukin-1beta and interleukin-1 receptor antagonist gene polymorphisms and gastric cancer: a meta-analysis. Cancer Epidemiol. Biomarkers Prev. 15, 1674–1687 10.1158/1055-9965.EPI-06-0189 16985030

[17] Fu W., Zhuo Z.-J., Chen Y.-C., Zhu J., Zhao Z., Jia W. (2017) NFKB1 -94insertion/deletion ATTG polymorphism and cancer risk: Evidence from 50 case-control studies. Oncogene 8, 9806–9822 10.18632/oncotarget.14190 PMC5354772 28039461

[18] Thorlund K E.J., Wetterslev J. (2011) User manual for trial sequential analysis (TSA). Copenhagen Trial Unit

[19] Wacholder S., Chanock S., Garcia-Closas M., El ghormli L. and Rothman N. (2004) Assessing the probability that a positive report is false: an approach for molecular epidemiology studies. JNCI J. Natl. Cancer Institute 96, 434–442 10.1093/jnci/djh075 PMC7713993 15026468

[20] He J., Wang M.Y., Qiu L.X., Zhu M.L., Shi T.Y., Zhou X.Y. (2013) Genetic variations of mTORC1 genes and risk of gastric cancer in an Eastern Chinese population. Mol. Carcinog. 52, E70–E79 10.1002/mc.22013 23423739

[21] Higgins J.P. and Thompson S.G. (2002) Quantifying heterogeneity in a meta-analysis. Stat. Med. 21, 1539–1558 10.1002/sim.1186 12111919

[22] Higgins J.P., Thompson S.G., Deeks J.J. and Altman D.G. (2003) Measuring inconsistency in meta-analyses. BMJ 327, 557–560 10.1136/bmj.327.7414.557 12958120 PMC192859

[23] Xie J., Zhang Y., Zhang Q., Han Y., Yin J., Pu R. (2013) Interaction of signal transducer and activator of transcription 3 polymorphisms with hepatitis B virus mutations in hepatocellular carcinoma. Hepatology 57, 2369–2377 10.1002/hep.26303 23386590

[24] Chanthra N., Payungporn S., Chuaypen N., Piratanantatavorn K., Pinjaroen N., Poovorawan Y. (2015) Single nucleotide polymorphisms in STAT3 and STAT4 and risk of hepatocellular carcinoma in Thai patients with chronic hepatitis B. Asian Pac. J. Cancer Prev. 16, 8405–8410 10.7314/APJCP.2015.16.18.8405 26745093

[25] Chanthra N., Payungporn S., Chuaypen N., Pinjaroen N., Poovorawan Y. and Tangkijvanich P. (2015) Association of single nucleotide polymorphism rs1053004 in signal transducer and activator of transcription 3 (STAT3) with susceptibility to hepatocellular carcinoma in Thai patients with chronic hepatitis B. Asian Pac. J. Cancer Prev. 16, 5069–5073 10.7314/APJCP.2015.16.12.5069 26163643

[26] Li M., Li F., Li N. (2018) Association of polymorphism rs1053005 in STAT3 with chronic hepatitis B virus infection in Han Chinese population. BMC Med. Genet. 19, 52 10.1186/s12881-018-0569-x 29609539 PMC5879595

[27] Fatemipour M., Arab Zadeh S.A.M., Molaei H., Geramizadeh B., Fatemipour B., Vahedi S.M. (2016) Study on the relationship of demographic characteristics of rs1053004 in STAT3 Gene in pationts with HCC following chronic HBV infection. Iran. J. Virol. 10, 40–47 10.21859/isv.10.2.3.40

[28] Chen K., Shi W., Xin Z., Wang H., Zhu X., Wu X. (2013) Replication of genome wide association studies on hepatocellular carcinoma susceptibility loci in a Chinese population. PLoS ONE 8, e77315 10.1371/journal.pone.0077315 24204805 PMC3810470

[29] Clark A., Gerlach F., Tong H., Hoan N.X., Song le H., Toan N.L. (2013) A trivial role of STAT4 variant in chronic hepatitis B induced hepatocellular carcinoma. Infect. Genet. Evol. 18, 257–261 10.1016/j.meegid.2013.05.025 23748017

[30] Kim L.H., Cheong H.S., Namgoong S., Kim J.O., Kim J.H., Park B.L. (2015) Replication of genome wide association studies on hepatocellular carcinoma susceptibility loci of STAT4 and HLA-DQ in a Korean population. Infect. Genet. Evol. 33, 72–76 10.1016/j.meegid.2015.04.013 25913043

[31] Liao Y., Cai B., Li Y., Chen J., Ying B., Tao C. (2015) Association of HLA-DP/DQ, STAT4 and IL-28B variants with HBV viral clearance in Tibetans and Uygurs in China. Liver Int. 35, 886–896 10.1111/liv.12643 25041342

[32] Chen W., Wang M., Zhang Z., Tang H., Zuo X., Meng X. (2015) Replication the association of 2q32.2–q32.3 and 14q32.11 with hepatocellular carcinoma. Gene 561, 63–67 10.1016/j.gene.2015.02.006 25665738

[33] Lu Y., Zhu Y., Peng J., Wang X., Wang F. and Sun Z. (2015) STAT4 genetic polymorphisms association with spontaneous clearance of hepatitis B virus infection. Immunol. Res. 62, 146–152 10.1007/s12026-015-8645-1 25829184

[34] El Sharkawy R., Thabet K., Lampertico P., Petta S., Mangia A., Berg T. (2018) A STAT4 variant increases liver fibrosis risk in Caucasian patients with chronic hepatitis B. Aliment. Pharmacol. Ther. 48, 564–573 10.1111/apt.14866 29963713

[35] Dick D.M. (2011) Gene-environment interaction in psychological traits and disorders. Annu. Rev. Clin. Psychol. 7, 383–409 10.1146/annurev-clinpsy-032210-104518 21219196 PMC3647367

[36] Sansone P. and Bromberg J. (2012) Targeting the interleukin-6/Jak/stat pathway in human malignancies. J. Clin. Oncol. 30, 1005–1014 10.1200/JCO.2010.31.8907 22355058 PMC3341105

[37] Kim H.Y., Jhun J.Y., Cho M.L., Choi J.Y., Byun J.K., Kim E.K. (2014) Interleukin-6 upregulates Th17 response via mTOR/STAT3 pathway in acute-on-chronic hepatitis B liver failure. J. Gastroenterol. 49, 1264–1273 10.1007/s00535-013-0891-1 24366287

[38] Chou C.H., Chen P.J., Jeng Y.M., Cheng A.L., Huang L.R. and Cheng J.C. (2009) Synergistic effect of radiation and interleukin-6 on hepatitis B virus reactivation in liver through STAT3 signaling pathway. Int. J. Radiat. Oncol. Biol. Phys. 75, 1545–1552 10.1016/j.ijrobp.2008.12.072 19327909

[39] Watford W.T., Hissong B.D., Bream J.H., Kanno Y., Muul L. and O’Shea J.J. (2004) Signaling by IL-12 and IL-23 and the immunoregulatory roles of STAT4. Immunol. Rev. 202, 139–156 10.1111/j.0105-2896.2004.00211.x 15546391

[40] Morinobu A., Gadina M., Strober W., Visconti R., Fornace A., Montagna C. (2002) STAT4 serine phosphorylation is critical for IL-12-induced IFN-gamma production but not for cell proliferation. Proc. Natl. Acad. Sci. U.S.A. 99, 12281–12286 10.1073/pnas.182618999 12213961 PMC129436

[41] Nishikomori R., Usui T., Wu C.Y., Morinobu A., O’Shea J.J. and Strober W. (2002) Activated STAT4 has an essential role in Th1 differentiation and proliferation that is independent of its role in the maintenance of IL-12R beta 2 chain expression and signaling. J. Immunol. 169, 4388–4398 10.4049/jimmunol.169.8.4388 12370372

[42] Remmers E.F., Plenge R.M., Lee A.T., Graham R.R., Hom G., Behrens T.W. (2007) STAT4 and the risk of rheumatoid arthritis and systemic lupus erythematosus. N. Engl. J. Med. 357, 977–986 10.1056/NEJMoa073003 17804842 PMC2630215

