# Supplementary material for: Association of STAT3 and STAT4 polymorphisms with susceptibility to chronic hepatitis B virus infection and risk of hepatocellular carcinoma: a meta-analysis
Source: Biosci Rep. 2019 Jun 20;39(6):BSR20190783. doi: 10.1042/BSR20190783 (PMC6616055; doi:10.1042/BSR20190783)
Supplement: Supplementary file 1 [file bsr20190783_Supp1.pdf]

	CC+CT		TT			Odds Ratio	
Study or Subgroup	Events	Total	Events	Total	Weight	M-H, Random, 95% CI	Year
Xie 2013	1179	1721	796	1249	40.1%	1.24 [1.06, 1.44]	2013
Chanthra 2015	316	445	128	205	24.0%	1.47 [1.04, 2.09]	2015
Fatemipour 2016	53	71	30	62	8.9%	3.14 [1.51, 6.52]	2016
Li 2018	236	373	190	320	27.0%	1.18 [0.87, 1.60]	2018
**Total (95% CI)**		**2610**		**1836**	**100.0%**	**1.38 [1.09, 1.76]**	
Total events	1784		1144				

Heterogeneity: $Tau^2 = 0.03$; $Chi^2 = 6.86$, df = 3 (P = 0.08); $I^2 = 56\%$

Test for overall effect: Z = 2.63 (P = 0.008)

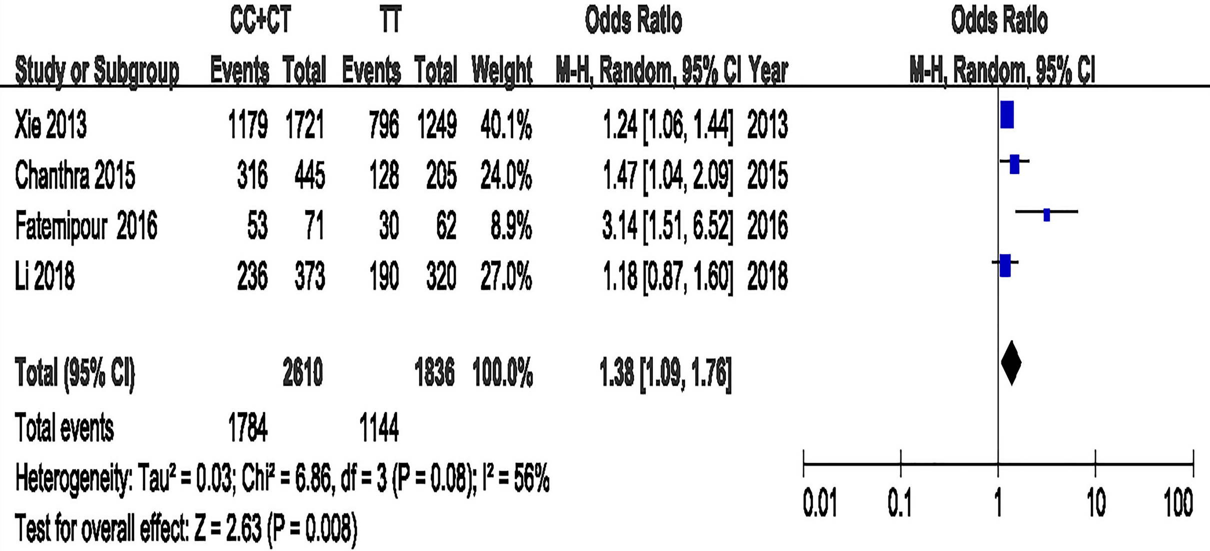

Study or Subgroup	CC Events	CC Total	CT+TT Events	CT+TT Total	Weight	Odds Ratio M-H, Fixed, 95% CI	Year
**1.1.1 CHB vs.HC+NC**							
Xie 2013	270	412	1705	2558	41.9%	0.95 [0.76, 1.18]	2013
Chanthra2015	82	112	362	538	8.6%	1.33 [0.84, 2.10]	2015
Fatemipour 2016	27	41	56	92	3.0%	1.24 [0.57, 2.68]	2016
Li 2018	51	70	375	623	5.3%	1.78 [1.02, 3.08]	2018
**Subtotal (95% CI)**		**635**		**3811**	**58.8%**	**1.10 [0.91, 1.31]**	
Total events	430		2498				
Heterogeneity: Chi² = 5.34, df = 3 (P = 0.15); I² = 44%							
Test for overall effect: Z = 0.99 (P = 0.32)							
**1.1.2 CHB with HCC vs.CHB without HCC（Southeast Asian）**							
Xie 2013	140	270	869	1705	29.4%	1.04 [0.80, 1.34]	2013
Chanthra2015	49	82	162	362	6.2%	1.83 [1.13, 2.99]	2015
Li 2018	23	51	164	375	5.5%	1.06 [0.59, 1.90]	2018
**Subtotal (95% CI)**		**403**		**2442**	**41.2%**	**1.16 [0.94, 1.43]**	
Total events	212		1195				
Heterogeneity: Chi² = 4.22, df = 2 (P = 0.12); I² = 53%							
Test for overall effect: Z = 1.37 (P = 0.17)							
**Total (95% CI)**		**1038**		**6253**	**100.0%**	**1.12 [0.98, 1.29]**	
Total events	642		3693				
Heterogeneity: Chi² = 9.74, df = 6 (P = 0.14); I² = 38%							
Test for overall effect: Z = 1.64 (P = 0.10)							

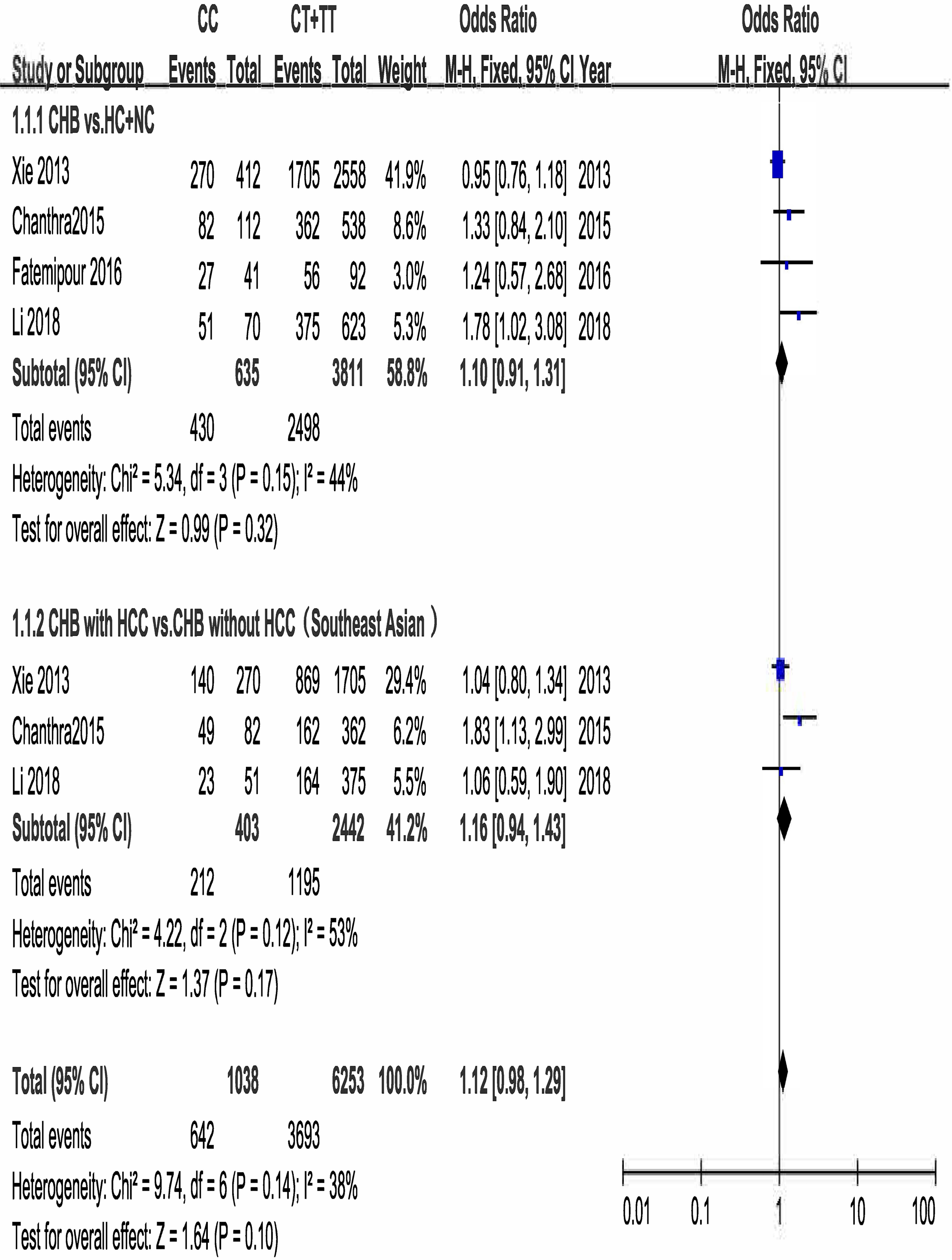

Study or Subgroup	CC+CT Events	CC+CT Total	TT Events	TT Total	Weight	Odds Ratio M-H, Fixed, 95% CI	Year
**1.1.1 CHB vs.HC+NC (Southeast Asian)**							
Xie 2013	1179	1721	796	1249	26.7%	1.24 [1.06, 1.44]	2013
Chanthra2015	316	445	128	205	4.7%	1.47 [1.04, 2.09]	2015
Li 2018	236	373	190	320	6.9%	1.18 [0.87, 1.60]	2018
**Subtotal (95% CI)**		**2539**		**1774**	**38.3%**	**1.26 [1.11, 1.43]**	
Total events	1731		1114				
Heterogeneity: Chi² = 1.00, df = 2 (P = 0.61); I² = 0%							
Test for overall effect: Z = 3.49 (P = 0.0005)							
**1.1.2 CHB with HCC vs.CHB without HCC**							
Xie 2013	598	1179	411	796	22.2%	0.96 [0.81, 1.15]	2013
Liao 2014	156	316	55	128	3.6%	1.29 [0.86, 1.96]	2014
Chanthra2015	23	53	10	30	0.7%	1.53 [0.60, 3.90]	2015
Fatemipour 2016	105	236	82	190	4.6%	1.06 [0.72, 1.55]	2016
**Subtotal (95% CI)**		**1784**		**1144**	**31.2%**	**1.03 [0.89, 1.19]**	
Total events	882		558				
Heterogeneity: Chi² = 2.40, df = 3 (P = 0.49); I² = 0%							
Test for overall effect: Z = 0.37 (P = 0.71)							
**1.1.3 CHB with HCC vs.CHB without HCC(Southeast Asian)**							
Xie 2013	598	1179	411	796	22.2%	0.96 [0.81, 1.15]	2013
Chanthra2015	156	316	55	128	3.6%	1.29 [0.86, 1.96]	2015
Li 2018	105	236	82	190	4.6%	1.06 [0.72, 1.55]	2018
**Subtotal (95% CI)**		**1731**		**1114**	**30.5%**	**1.02 [0.87, 1.18]**	
Total events	859		548				
Heterogeneity: Chi² = 1.68, df = 2 (P = 0.43); I² = 0%							
Test for overall effect: Z = 0.22 (P = 0.82)							
**Total (95% CI)**		**6054**		**4032**	**100.0%**	**1.11 [1.02, 1.21]**	
Total events	3472		2220				
Heterogeneity: Chi² = 10.97, df = 9 (P = 0.28); I² = 18%							
Test for overall effect: Z = 2.55 (P = 0.01)							

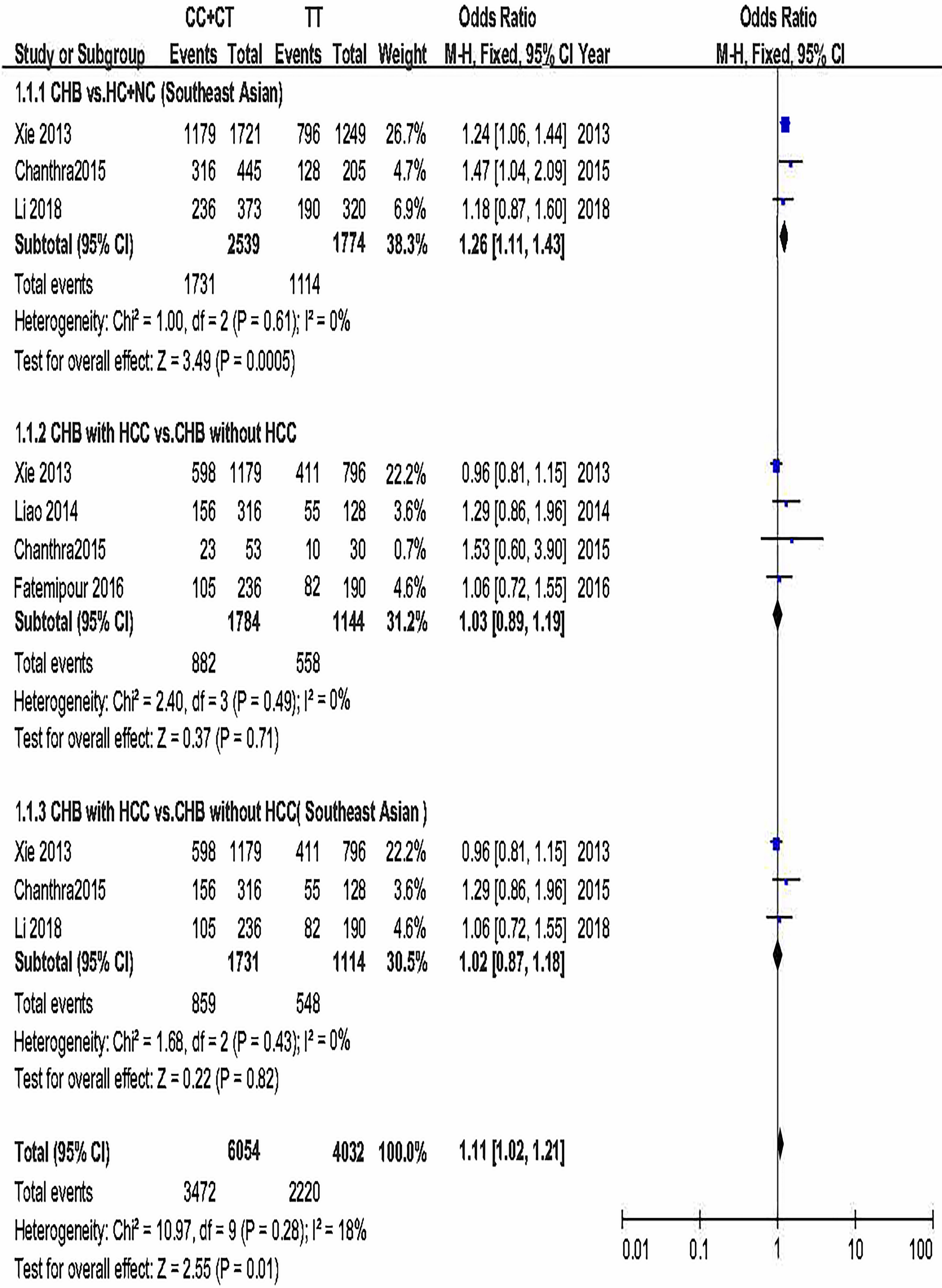

Study or Subgroup	CC Events	CC Total	CT+CC Events	CT+CC Total	Weight	Odds Ratio M-H, Random, 95% CI	Year
**1.1.1 CHB vs.HC+NC（Southeast Asian）**							
Xie 2013	270	412	1705	2558	21.6%	0.95 [0.76, 1.18]	2013
Chanthra2015	82	112	362	538	14.8%	1.33 [0.84, 2.10]	2015
Li 2018	51	70	375	623	12.5%	1.78 [1.02, 3.08]	2018
**Subtotal (95% CI)**		**594**		**3719**	**48.9%**	**1.23 [0.85, 1.77]**	
Total events	403		2442				
Heterogeneity: Tau² = 0.07; Chi² = 5.22, df = 2 (P = 0.07); I² = 62%							
Test for overall effect: Z = 1.08 (P = 0.28)							
**1.1.2 CHB with HCC vs.CHB without HCC**							
Xie 2013	140	270	869	1705	20.5%	1.04 [0.80, 1.34]	2013
Chanthra2015	49	82	162	362	14.0%	1.83 [1.13, 2.99]	2015
Fatemipour 2016	5	27	28	56	5.0%	0.23 [0.08, 0.69]	2016
Li 2018	23	51	164	375	11.7%	1.06 [0.59, 1.90]	2018
**Subtotal (95% CI)**		**430**		**2498**	**51.1%**	**0.98 [0.58, 1.67]**	
Total events	217		1223				
Heterogeneity: Tau² = 0.20; Chi² = 12.30, df = 3 (P = 0.006); I² = 76%							
Test for overall effect: Z = 0.06 (P = 0.95)							
**Total (95% CI)**		**1024**		**6217**	**100.0%**	**1.14 [0.86, 1.50]**	
Total events	620		3665				
Heterogeneity: Tau² = 0.08; Chi² = 17.50, df = 6 (P = 0.008); I² = 66%							
Test for overall effect: Z = 0.91 (P = 0.36)							

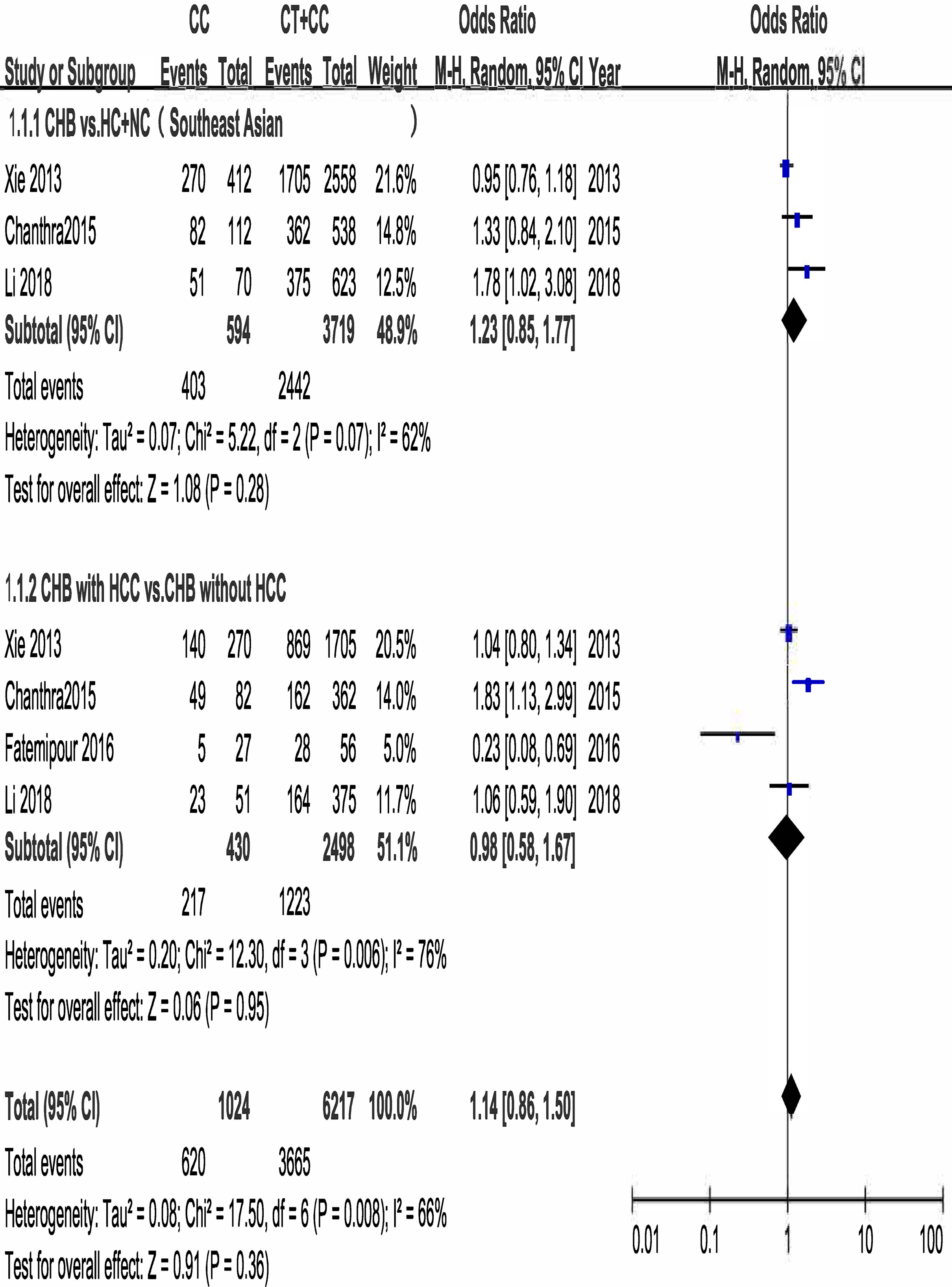

Study or Subgroup	G Events	G Total	C Events	C Total	Weight	Odds Ratio M-H, Fixed, 95% CI	Year
**1.1.1 CHB VS.HC**							
Xie 2013	1862	2792	1982	3070	83.5%	1.10 [0.99, 1.22]	2013
Chanthra 2015	424	625	360	539	16.5%	1.05 [0.82, 1.34]	2015
**Subtotal (95% CI)**		**3417**		**3609**	**100.0%**	**1.09 [0.99, 1.20]**	
Total events	2286		2342				
Heterogeneity: Chi² = 0.12, df = 1 (P = 0.73); I² = 0%							
Test for overall effect: Z = 1.72 (P = 0.08)							
**1.1.2 CHB with HCC vs.CHB without HCC**							
Xie 2013	1000	1862	1026	1982	82.7%	1.08 [0.95, 1.23]	2013
Chanthra2015	209	424	175	360	17.3%	1.03 [0.78, 1.36]	2015
**Subtotal (95% CI)**		**2286**		**2342**	**100.0%**	**1.07 [0.95, 1.20]**	
Total events	1209		1201				
Heterogeneity: Chi² = 0.10, df = 1 (P = 0.75); I² = 0%							
Test for overall effect: Z = 1.18 (P = 0.24)							

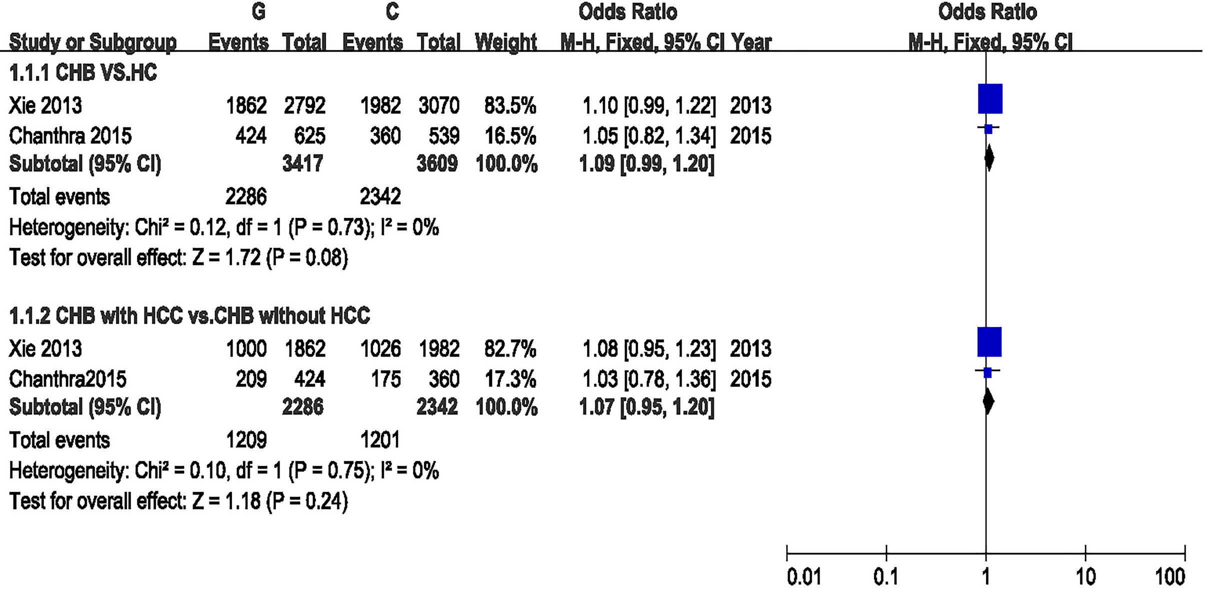

Study or Subgroup	GG+GC Events	GG+GC Total	CC Events	CC Total	Weight	Odds Ratio M-H, Fixed, 95% CI	Year
**1.1.1 CHB VS.HC**							
Xie 2013	1412	2127	510	804	85.3%	1.14 [0.96, 1.35]	2013
Chanthra2015	308	456	84	126	14.7%	1.04 [0.68, 1.58]	2015
**Subtotal (95% CI)**		**2583**		**930**	**100.0%**	**1.12 [0.96, 1.32]**	
Total events	1720		594				
Heterogeneity: Chi² = 0.15, df = 1 (P = 0.70); I² = 0%							
Test for overall effect: Z = 1.46 (P = 0.14)							
**1.1.2 CHB with HCC vs.CHB without HCC**							
Xie 2013	748	1412	265	510	85.6%	1.04 [0.85, 1.28]	2013
Chanthra2015	153	308	39	84	14.4%	1.14 [0.70, 1.85]	2015
**Subtotal (95% CI)**		**1720**		**594**	**100.0%**	**1.06 [0.88, 1.27]**	
Total events	901		304				
Heterogeneity: Chi² = 0.11, df = 1 (P = 0.74); I² = 0%							
Test for overall effect: Z = 0.57 (P = 0.57)							

Odds Ratio
M-H, Fixed, 95% CI

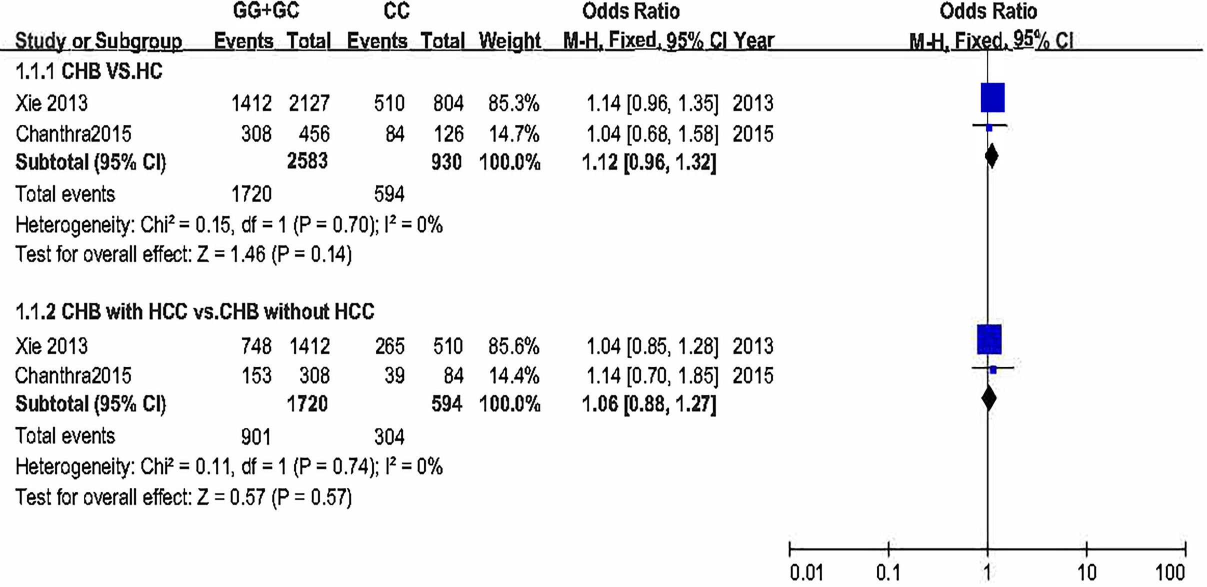

Study or Subgroup	GG Events	GG Total	GC+CC Events	GC+CC Total	Weight	Odds Ratio M-H, Fixed, 95% CI	Year	Odds Ratio M-H, Fixed, 95% CI
**1.1.1 CHB VS.HC**								
Xie 2013	450	665	1472	2266	81.1%	1.13 [0.94, 1.36]	2013	
Chanthra2015	116	169	276	413	18.9%	1.09 [0.74, 1.60]	2015	
**Subtotal (95% CI)**		**834**		**2679**	**100.0%**	**1.12 [0.95, 1.32]**		
Total events	566		1748					
Heterogeneity: Chi² = 0.03, df = 1 (P = 0.86); I² = 0%								
Test for overall effect: Z = 1.35 (P = 0.18)								
**1.1.2 CHB with HCC vs.CHB without HCC**								
Xie 2013	252	450	761	1472	79.0%	1.19 [0.96, 1.47]	2013	
Chanthra2015	56	116	136	276	21.0%	0.96 [0.62, 1.48]	2015	
**Subtotal (95% CI)**		**566**		**1748**	**100.0%**	**1.14 [0.94, 1.38]**		
Total events	308		897					
Heterogeneity: Chi² = 0.75, df = 1 (P = 0.39); I² = 0%								
Test for overall effect: Z = 1.36 (P = 0.17)								

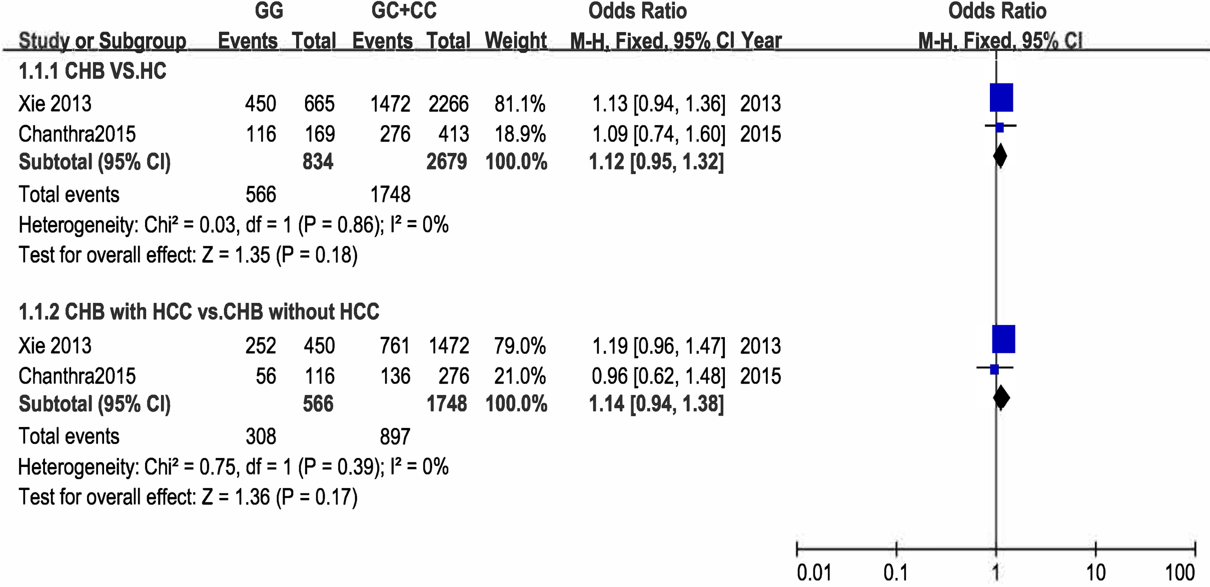

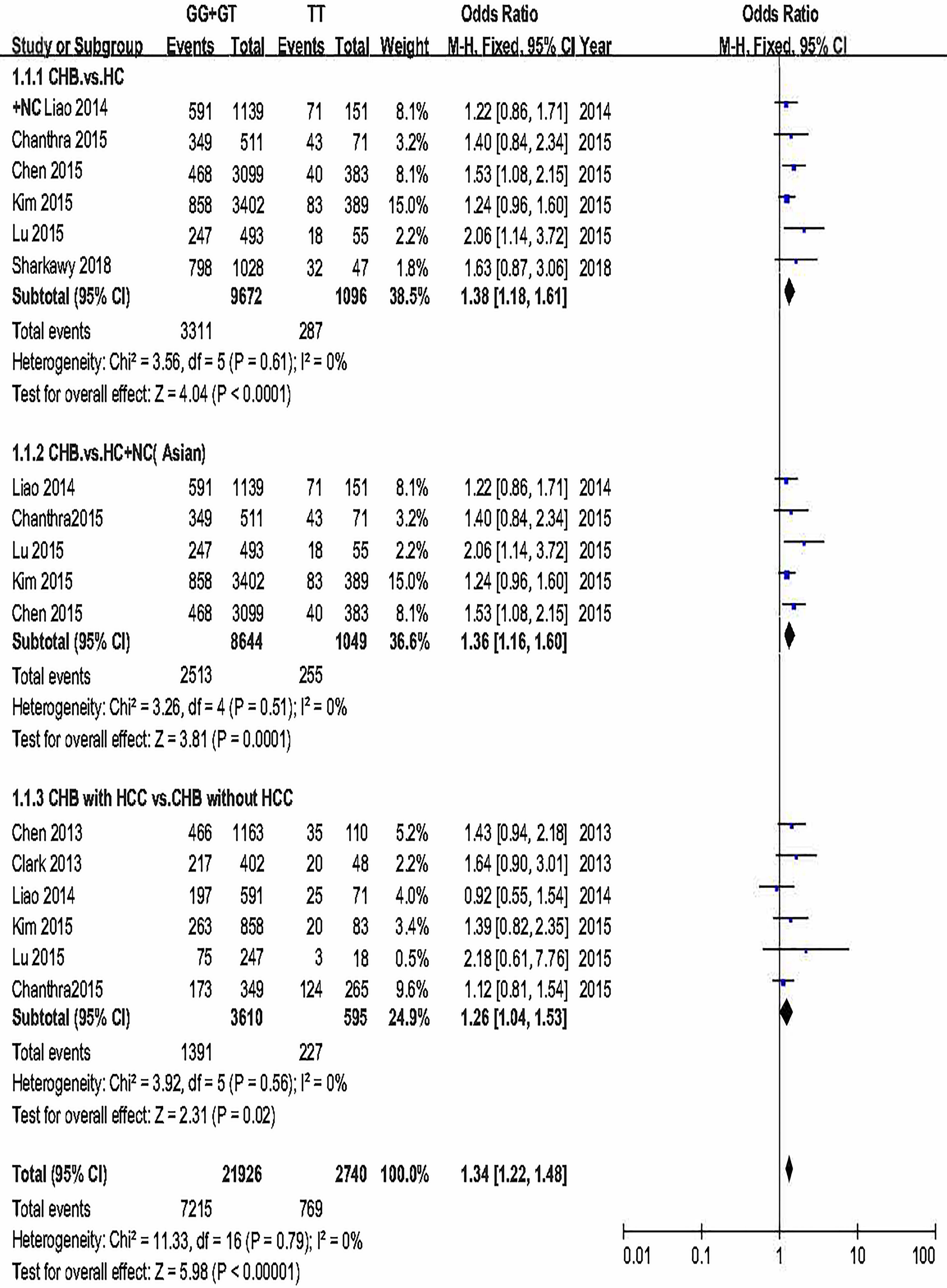

GG+GT
TT
Odds Ratio
Odds Ratio
Study or Subgroup Events Total Events Total Weight M-H, Fixed, 95% CI Year
M-H, Fixed, 95% CI
1.1.1 CHB.vs.HC
+NC Liao 2014 591 1139 71 151 8.1% 1.22 [0.86, 1.71] 2014
Chanthra 2015 349 511 43 71 3.2% 1.40 [0.84, 2.34] 2015
Chen 2015 468 3099 40 383 8.1% 1.53 [1.08, 2.15] 2015
Kim 2015 858 3402 83 389 15.0% 1.24 [0.96, 1.60] 2015
Lu 2015 247 493 18 55 2.2% 2.06 [1.14, 3.72] 2015
Sharkawy 2018 798 1028 32 47 1.8% 1.63 [0.87, 3.06] 2018
Subtotal (95% CI) 9672 1096 38.5% 1.38 [1.18, 1.61]
Total events 3311 287
Heterogeneity: Chi² = 3.56, df = 5 (P = 0.61); I² = 0%
Test for overall effect: Z = 4.04 (P < 0.0001)
1.1.2 CHB.vs.HC+NC(Asian)
Liao 2014 591 1139 71 151 8.1% 1.22 [0.86, 1.71] 2014
Chanthra2015 349 511 43 71 3.2% 1.40 [0.84, 2.34] 2015
Lu 2015 247 493 18 55 2.2% 2.06 [1.14, 3.72] 2015
Kim 2015 858 3402 83 389 15.0% 1.24 [0.96, 1.60] 2015
Chen 2015 468 3099 40 383 8.1% 1.53 [1.08, 2.15] 2015
Subtotal (95% CI) 8644 1049 36.6% 1.36 [1.16, 1.60]
Total events 2513 255
Heterogeneity: Chi² = 3.26, df = 4 (P = 0.51); I² = 0%
Test for overall effect: Z = 3.81 (P = 0.0001)
1.1.3 CHB with HCC vs.CHB without HCC
Chen 2013 466 1163 35 110 5.2% 1.43 [0.94, 2.18] 2013
Clark 2013 217 402 20 48 2.2% 1.64 [0.90, 3.01] 2013
Liao 2014 197 591 25 71 4.0% 0.92 [0.55, 1.54] 2014
Kim 2015 263 858 20 83 3.4% 1.39 [0.82, 2.35] 2015
Lu 2015 75 247 3 18 0.5% 2.18 [0.61, 7.76] 2015
Chanthra2015 173 349 124 265 9.6% 1.12 [0.81, 1.54] 2015
Subtotal (95% CI) 3610 595 24.9% 1.26 [1.04, 1.53]
Total events 1391 227
Heterogeneity: Chi² = 3.92, df = 5 (P = 0.56); I² = 0%
Test for overall effect: Z = 2.31 (P = 0.02)
Total (95% CI) 21926 2740 100.0% 1.34 [1.22, 1.48]
Total events 7215 769
Heterogeneity: Chi² = 11.33, df = 16 (P = 0.79); I² = 0%
Test for overall effect: Z = 5.98 (P < 0.00001)
0.01 0.1 1 10 100

Study or Subgroup	GG Events	GG Total	GT+TT Events	GT+TT Total	Weight	Odds Ratio M-H, Fixed, 95% CI	Year
**1.1.1 CHB vs.NC+HC**							
Liao 2013	103	194	95	214	1.9%	1.42 [0.96, 2.09]	2013
Liao 2013	194	403	189	457	4.0%	1.32 [1.00, 1.72]	2013
Liao 2014	294	572	368	718	7.0%	1.01 [0.81, 1.25]	2014
Kim 2015	494	1787	447	2004	13.4%	1.33 [1.15, 1.54]	2015
Lu 2015	122	236	143	312	2.6%	1.26 [0.90, 1.77]	2015
Chen 2015	257	1555	251	1927	8.2%	1.32 [1.10, 1.60]	2015
Chanthra2015	170	232	222	350	2.1%	1.58 [1.10, 2.27]	2015
Sharkawy 2018	546	693	284	392	3.4%	1.41 [1.06, 1.88]	2018
**Subtotal (95% CI)**		**5672**		**6374**	**42.6%**	**1.29 [1.19, 1.41]**	
Total events	2180		1999				
Heterogeneity: $Chi^2 = 7.01$, df = 7 (P = 0.43); $I^2 = 0\%$							
Test for overall effect: Z = 6.04 (P < 0.00001)							
**1.1.2 CHB vs.NC+HC(Asian)**							
Liao 2013	103	194	95	214	1.9%	1.42 [0.96, 2.09]	2013
Liao 2013	194	403	189	457	4.0%	1.32 [1.00, 1.72]	2013
Liao 2014	294	572	368	718	7.0%	1.01 [0.81, 1.25]	2014
Lu 2015	122	236	143	312	2.6%	1.26 [0.90, 1.77]	2015
Kim 2015	494	1787	447	2004	13.4%	1.33 [1.15, 1.54]	2015
Chanthra2015	170	232	222	350	2.1%	1.58 [1.10, 2.27]	2015
Chen 2015	257	1555	251	1927	8.2%	1.32 [1.10, 1.60]	2015
**Subtotal (95% CI)**		**4979**		**5982**	**39.2%**	**1.28 [1.18, 1.40]**	
Total events	1634		1715				
Heterogeneity: $Chi^2 = 6.61$, df = 6 (P = 0.36); $I^2 = 9\%$							
Test for overall effect: Z = 5.60 (P < 0.00001)							
**1.1.3 CHB with HCC vs.CHB without HCC**							
Clark 2013	117	203	127	247	2.1%	1.29 [0.88, 1.87]	2013
Chen 2013	249	619	252	654	6.4%	1.07 [0.86, 1.34]	2013
Liao 2014	104	294	118	368	3.0%	1.16 [0.84, 1.60]	2014
Chanthra 2015	87	170	105	222	2.0%	1.17 [0.78, 1.74]	2015
Kim 2015	160	494	123	447	3.8%	1.26 [0.95, 1.67]	2015
Lu 2015	45	122	33	143	0.8%	1.95 [1.14, 3.33]	2015
**Subtotal (95% CI)**		**1902**		**2081**	**18.2%**	**1.20 [1.06, 1.37]**	
Total events	762		758				
Heterogeneity: $Chi^2 = 4.40$, df = 5 (P = 0.49); $I^2 = 0\%$							
Test for overall effect: Z = 2.78 (P = 0.005)							
**Total (95% CI)**		**12553**		**14437**	**100.0%**	**1.27 [1.20, 1.34]**	
Total events	4576		4472				
Heterogeneity: $Chi^2 = 18.91$, df = 20 (P = 0.53); $I^2 = 0\%$							
Test for overall effect: Z = 8.64 (P < 0.00001)							

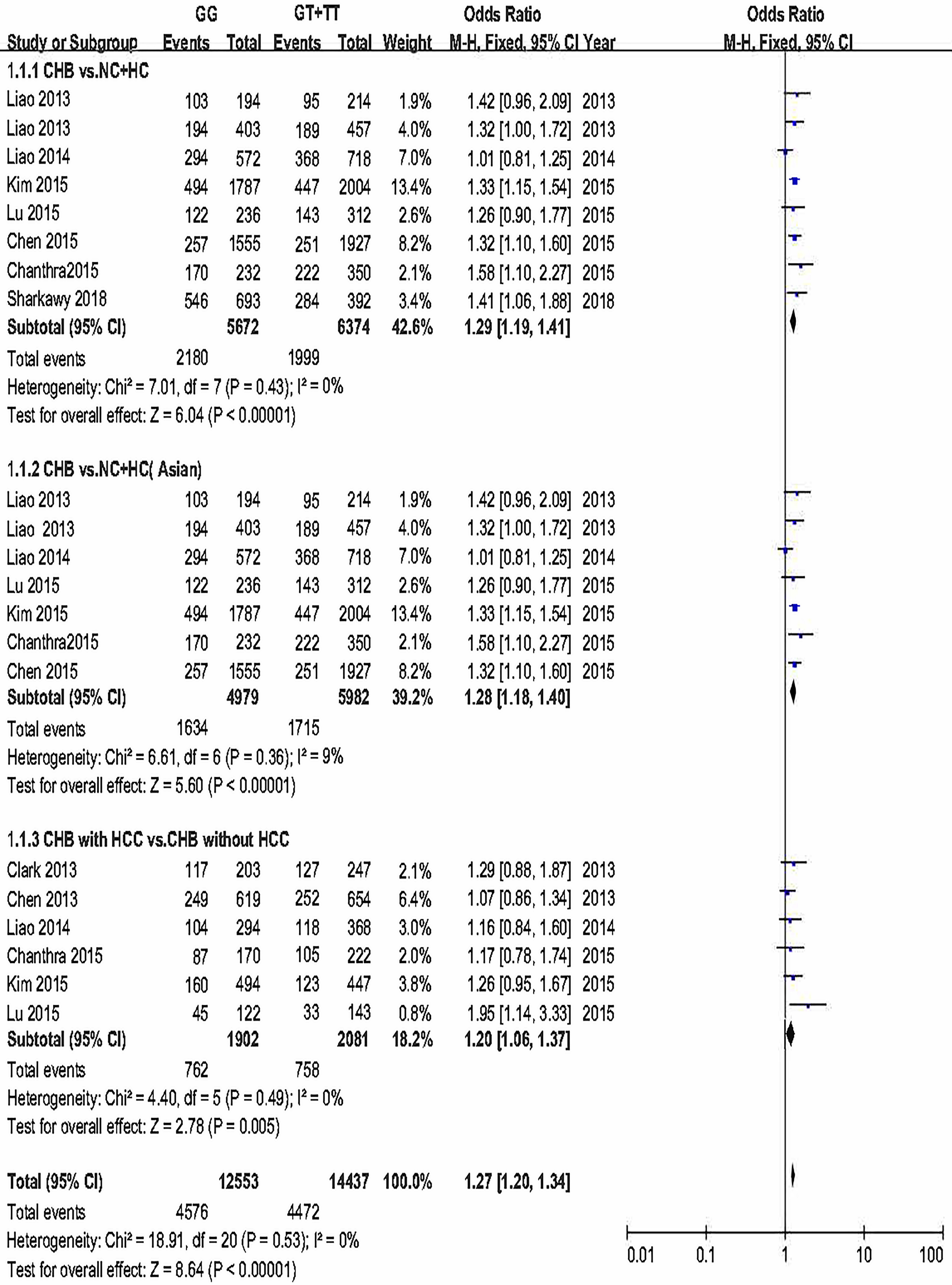

Supplemental Table S. Score of quality assessment

Criteria	Score
Representativeness of case	
Selected from population cancer registry	2
Selected from hospital	1
No method of selection described	0
Representativeness of control	
Population-based	3
Blood donors	2
Hospital-based	1
Not described	0
Ascertainment of NHL case	
Histopathologic confirmation	2
by patient medical record	1
Not described	0
Control selection	
Controls matched with cases by age and sex	2
Controls matched with cases only by age or by sex	1
Not matched or not descried	0
Genotyping examination	
Genotyping done blindly and quality control	2
Only genotyping done blindly or quality control	1
Unblinded and without quality control	0
HWE	
HWE in the control group	1
HWD in the control group or not mentioned	0
Total sample size	
> 1000	3
501 - 1000	2
201 - 500	1
≤ 200	0
